# circCIMT Silencing Promotes Cadmium‐Induced Malignant Transformation of Lung Epithelial Cells Through the DNA Base Excision Repair Pathway

**DOI:** 10.1002/advs.202206896

**Published:** 2023-02-22

**Authors:** Meizhen Li, Wei Chen, Jinjin Cui, Qiuyi Lin, Yufei Liu, Huixian Zeng, Qiuhan Hua, Yihui Ling, Xiaodi Qin, Yindai Zhang, Xueqi Li, Tianshu Lin, Lihua Huang, Yiguo Jiang

**Affiliations:** ^1^ State Key Laboratory of Respiratory Disease The First Affiliated Hospital of Guangzhou Medical University Guangzhou 510120 P. R. China; ^2^ Institute for Chemical Carcinogenesis Guangzhou Medical University Guangzhou 511436 P. R. China; ^3^ School of Public Health Baotou Medical College Baotou 014030 P. R. China

**Keywords:** cadmium, circCIMT, DNA damage response, malignant transformation of cells

## Abstract

Changes in gene expression in lung epithelial cells are detected in cancer tissues during exposure to pollutants, highlighting the importance of gene‐environmental interactions in disease. Here, a Cd‐induced malignant transformation model in mouse lungs and bronchial epithelial cell lines is constructed, and differences in the expression of non‐coding circRNAs are analyzed. The migratory and invasive abilities of Cd‐transformed cells are suppressed by circCIMT. A significant DNA damage response is observed after exposure to Cd, which increased further following circCIMT‐interference. It is found that APEX1 is significantly down‐regulated following Cd exposure. Furthermore, it is demonstrated that circCIMT bound to APEX1 during Cd exposure to mediate the DNA base excision repair (BER) pathway, thereby reducing DNA damage. In addition, simultaneous knockdown of both circCIMT and APEX1 promotes the expression of cancer‐related genes and malignant transformation after long‐term Cd exposure. Overall, these findings emphasis the importance of genetic‐epigenetic interactions in chemical‐induced cancer transformation.

## Graphical abstract

The regulation of DNA damage by circRNA is closely associated with cell malignant transformation after long‐term exposure to Cd. This study shows that circCIMT is down‐regulated after Cd exposure. Consequently, circCIMT binding to APEX1 is decreased, and the expression of BER complex proteins is regulated, thus increasing DNA damage and controlling cell malignant transformation after Cd exposure.

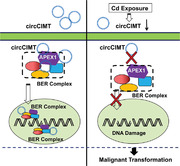

## 1 Introduction

Exposure of human lung tissue to Cadmium (Cd) is mainly through the inhalation of cigarette smoke and airborne particulate matter. Cd is a strong carcinogen and is classified as a Group I human lung carcinogen by the International Agency for Research on Cancer (IARC).^[^ ^1^ ^]^ Cd spreads through the surface of cigarette smoke and enters the host during respiration.^[^ ^2^ ^]^ Understanding the mechanism of lung cancer development after Cd exposure may be beneficial for the early detection and intervention of tumors. Changes in gene expression during heavy metal exposure are involved in mediating the harmful reactions of these environmental factors.^[^ ^3^ ^]^ However, it is short‐sighted to define the occurrence of malignant tumors only in terms of genetics. Epigenetics also play an important role in this process, and the relationship between genetic‐epigenetic interactions in chemical‐induced disease requires further elucidation.

Circular RNAs (circRNAs) are a type of non‐coding RNA that form a closed circular structure at the 5 “and 3” ends, and regulate gene expression in multiple ways, including transcriptional and post‐transcriptional regulation.^[^ ^4^, ^5^, ^6^ ^]^ circRNAs have a highly stable and tissue‐specific expression pattern, and are involved in many cell functions and disease processes. However, the functions of most of the thousands of unique circRNAs discovered to date remain unknown. circRNAs have different functions and biogenetic mechanisms and are associated with diverse cancers.^[^ ^7^ ^]^ Since abnormal expression of circRNAs is ubiquitous in the occurrence and development of lung cancer, circRNAs may act as carcinogenic or anticancer agents by affecting cellular functions.^[^ ^6^ ^]^ During the development of lung cancer, abnormal expression of circRNAs leads to the activation of multiple signaling pathways.^[^ ^8^, ^9^ ^]^ In addition, functional defects in the DNA damage repair system drive the progression of tumors through heightened genetic instability that cannot be repaired correctly, increasing pressure on the DNA replication machinery, increasing the mutation rate and triggering genomic changes.^[^ ^10^, ^11^ ^]^ Disruption of the DNA repair pathway aggravates the degree of DNA damage, which is a key factor promoting tumorigenesis during heavy metal exposure.^[^ ^12^ ^]^ However, it is unclear whether circRNAs can affect lung cancer progression through DNA damage repair during Cd exposure.

DNA base excision repair (BER) is a highly conserved DNA damage repair pathway that is critical for the repair of different types of endogenous and exogenous DNA base damage.^[^ ^13^, ^14^ ^]^ The BER pathway is mediated through the joint participation of multiple DNA glycosylases, such as AP endonuclease (APEX1), DNA ligase I (LIG1) or DNA ligase III (LIG3), poly (ADP‐ribose) polymerase‐1 (PARP1), and X‐ray repair cross‐complementing protein (XRCC1).^[^ ^13^, ^14^, ^15^, ^16^ ^]^ For example, APEX1 and XRCC1 have been shown to participate in damage recognition and base repair at the site of the DNA damage.^[^ ^17^ ^]^ Based on the important functions of both circRNAs and DNA damage, we speculated that circRNAs play a role in regulating DNA damage during the development of Cd‐induced lung cancer. Here, we constructed a chronic Cd‐induced malignant transformation model in mouse lungs and bronchial epithelial cell lines, and found that circRNA expression profiles changed significantly. Specifically, circCIMT negatively regulated Cd‐induced malignant transformation. We further demonstrated that circCIMT interacted with APEX1 to suppress DNA damage through the BER pathway repair. Our study highlights the role of epigenetics in mediating genetic damage during Cd‐induced lung cancer.

## 2 Results and Discussion

### 2.1 Chronic Low‐Dose Cd Exposure Results in the Development of Malignant Lesions in Mouse Lungs and Malignant Transformation of Bronchial Epithelial Cells

**Figure 1:**
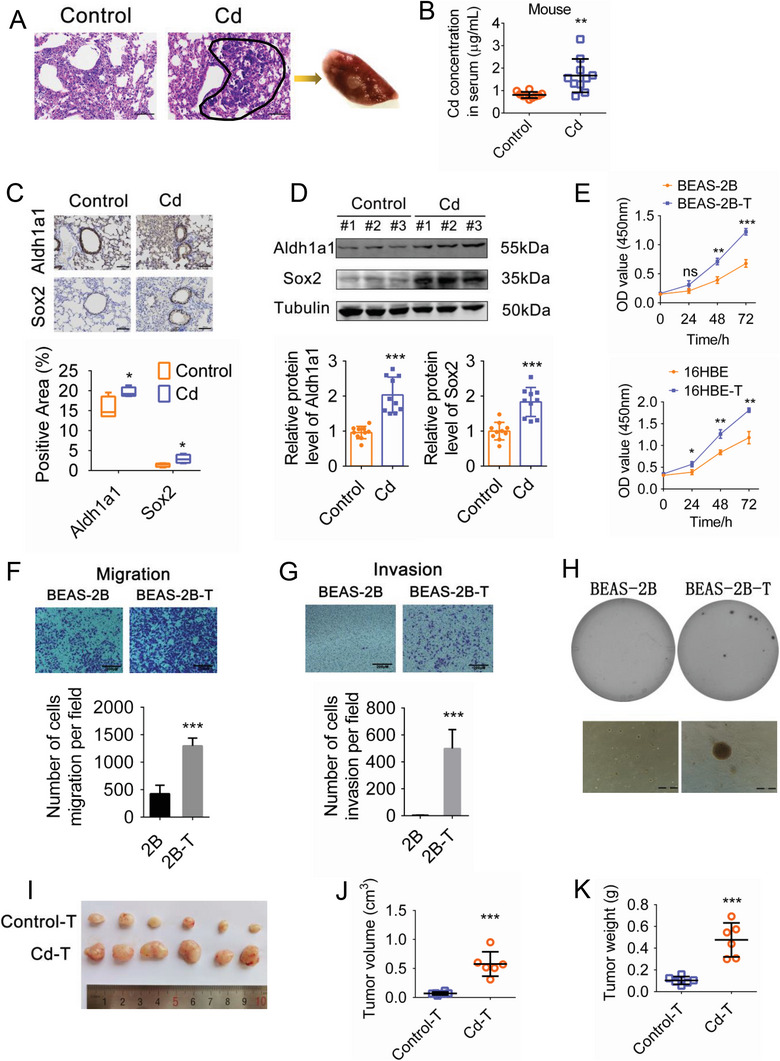
Chronic low‐dose Cd exposure results in malignant lesions in the mouse lung and malignant transformation of bronchial epithelial cells. A) Representative H&E staining of lung lesions in untreated and Cd‐treated mice after 45 weeks. Scale bar = 100 µM. B) Concentration of Cd in mouse serum. n (Control) = 10, n (Cd) = 10. C) Immunohistochemical analysis of Aldh1a1 and Sox2 expression levels in the lung tissue of untreated and Cd‐treated mice. Scale bar = 100 µM. D) Expression levels of Aldh1a1 and Sox2 in the lung tissue of untreated and Cd‐treated mice were assessed by Western blot analysis. n (Control) = 10, n (Cd) = 10. E) Relative levels of proliferation of BEAS‐2B (above) and 16HBE (below) Cd‐transformed cells were determined using the CCK8 assay. F) The Transwell assay was used to determine levels of migration in Cd‐transformed cells. G) The Transwell assay was used to assess levels of invasion in Cd‐transformed cells. H) Sphere formation with unanchored growth of Cd‐transformed cells. I–K) The tumor volumes and weights of Cd‐induced tumors in a nude mouse xenograft model. *p < 0.05, **p < 0.01, ***p < 0.001.

In order to examine the impact of Cd on malignant transformation, we established a chronic Cd exposure model in vivo by exposing mice to Cd via nose and mouth inhalation (Figure S1A, Supporting Information). On the basis of real‐time exposure data in the human body,^[^ ^2^ ^]^ we calculated that the equivalent real‐time exposure concentration in mice would be 0.640 mg m⁻^3^. After 45 weeks of Cd exposure, pathological examination indicated areas of large nucleated cells, large areas of basophilic cells, and abnormal hyperplasia in the lung tissue (**Figure** **1**A). In addition, high levels of Cd were detected in the serum (Figure 1B). Previous studies have demonstrated that cancer stem cells (CSCs) have the ability to promote the development of the primary tumor.^[^ ^18^ ^]^ Here, we used immunohistochemical staining and western blot analysis to examine the expression of Aldh1a1 and Sox2, molecular markers of CSCs. We found that both Aldh1a1 and Sox2 expression levels were significantly increased in the lung following Cd exposure (Figure 1C,D), suggesting that malignant transformation occurs in the lung tissue after prolonged Cd inhalation.

The normal bronchial epithelial cell lines, BEAS‐2B and 16HBE, were selected to construct an in vitro Cd‐induced malignant transformation model in cells. For 16HBE cells, the minimum exposure (10 µM) was calculated by estimating the accumulated concentration of Cd in the blood of humans exposed to cigarettes for 1 year.^[^ ^2^ ^]^ Based on the cell viability data obtained from the CCK8 assay (Figure S1B,C, Supporting Information), our selected exposure concentrations were designated as low (1 µM and 10 µM), medium (2 µM and 20 µM) and high (4 µM and 40 µM). A Cd‐induced malignant transformation cellular model was constructed to examine the effects of chronic low‐dose exposure on cell transformation by continuously exposing BEAS‐2B and 16HBE cells to 1 µM and 10 µM Cd, respectively. After 65 weeks Cd exposure, enhanced proliferative and migratory abilities were observed in Cd‐transformed cells, as well as increased invasion, and sphere formation with unanchored growth (Figure 1E–H). In vivo, the tumor volumes and weights of Cd‐transformed cells in nude mice were significantly larger than those in the control group (Figure 1I–K). Taken together, our in vitro and in vivo results indicate that chronic low‐dose Cd exposure can directly promote malignant transformation of bronchial epithelial cells and the development of malignant lesions in the mouse lung.

### 2.2 The Expression and Function of circCIMT in a Cd‐Induced Malignant Transformation Cellular Model

**Figure 2:**
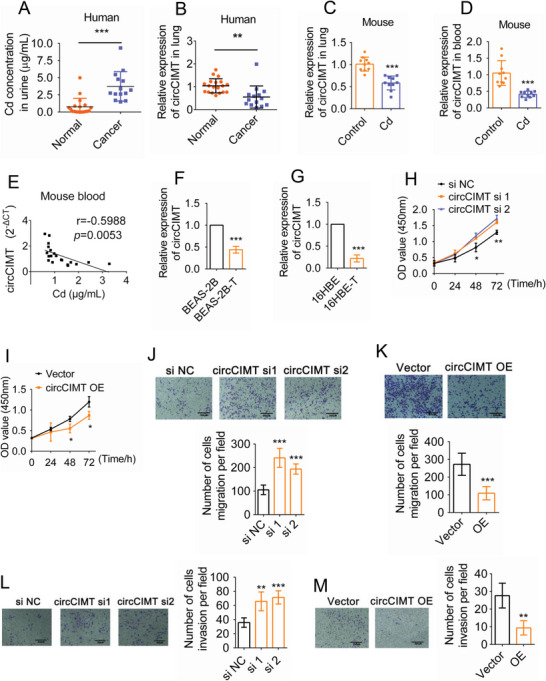
Expression and function of circCIMT in a Cd‐induced model of malignant transformation. A) Concentration of Cd in human urine. n (normal) = 19, n (cancer) = 14. B) circCIMT expression in human normal and cancer lung tissues. n (normal) = 19, n (cancer) = 14. C,D) q‐PCR was used to determine circCIMT expression levels in the lung tissue (C) and blood (D) of untreated and Cd‐treated mice. n (Control) = 10, n (Cd) = 10. E) Correlation between circCIMT (2^−ΔCT^) levels and Cd concentration in mouse blood. F,G) circCIMT expression levels were examined in Cd‐transformed BEAS‐2B (F) and 16HBE (G) cells. H,I) The CCK8 assay was used to examine the effects of circCIMT knockdown and overexpression on the proliferative abilities of Cd‐transformed BEAS‐2B cells. J,K) The Transwell assay was used to determine the effects of circCIMT knockdown (J) and overexpression (K) on the migration of Cd‐transformed BEAS‐2B cells. L,M) The Transwell assay was used to determine the effects of circCIMT knockdown (L) and overexpression (M) on the invasive abilities of Cd‐transformed BEAS‐2B cells. *p < 0.05, **p < 0.01, ***p < 0.001.

Our central hypothesis is that circRNAs influence Cd‐induced malignant transformation. To explore this hypothesis, we analyzed the expression profiles of circRNAs by whole transcriptome sequencing after 10 weeks exposure to 1 µM Cd. A total of 281 circRNAs were identified in BEAS‐2B cells (Table S1, Supporting Information). A heatmap was performed on the 40 genes that showed the most significant up‐ and down‐regulation (| fold change | ≥ 2, *p* value < 0.05) (Figure S2A,B, Supporting Information). qPCR analysis revealed that novel_circ_004401 was the most significantly down‐regulated circRNA in cells after Cd exposure for 10 weeks (Figure S2C, Supporting Information). The novel_circ_004401 was named circCIMT (circRNA involved in Cd‐Induced Malignant Transformation). circCIMT was 253 nt in length, and derived from exons 2 and 3 on the EZH2 gene located on chromosome 7. Furthermore, circCIMT was resistant to digestion with RNase R and junction site specificity (Figure s2d–f, Supporting Information). Elevated Cd levels were observed in human urine obtained from lung cancer patients, while decreased circCIMT expression was found in lung cancer tissue (**Figure** **2**A,B). Similarly, lower circCIMT expression was observed in the mouse lung tissue and blood following Cd exposure (Figure 2C,D). Furthermore, a negative correlation between circCIMT and Cd concentration was observed (Figure 2E). Finally, circCIMT expression levels were also found to be significantly reduced in Cd‐transformed BEAS‐2B and 16HBE cells (Figure 2F,G).

Next, we sought to explore the function of circCIMT in Cd‐transformed cells and identify genes with functional significance, to provide a basis for further research on the preventive effects of circCIMT on malignant transformation. An interference and overexpression model of circCIMT was constructed (Figure S2G,H, Supporting Information). We examined the effects of silencing and overexpressing circCIMT on Cd‐transformed cells. We found that circCIMT‐interference promoted the growth of Cd‐transformed BEAS‐2B cells, while overexpression of circCIMT inhibited cell growth (Figure 2H,I). In addition, the migratory and invasive abilities of Cd‐transformed BEAS‐2B cells were enhanced after circCIMT interference, while overexpressing circCIMT led to a reduction in migration and invasion (Figure 2J–M). Finally, circCIMT knockdown increased the amplification ability and anchorage‐independent growth of Cd‐transformed BEAS‐2B cells, while circCIMT overexpression had the opposite effect (Figure S2I,J, Supporting Information). Taken together, our data suggest that circCIMT negatively regulates the development of Cd‐transformed cells.

### 2.3 circCIMT Suppresses Cd‐Induced DNA Damage

**Figure 3:**
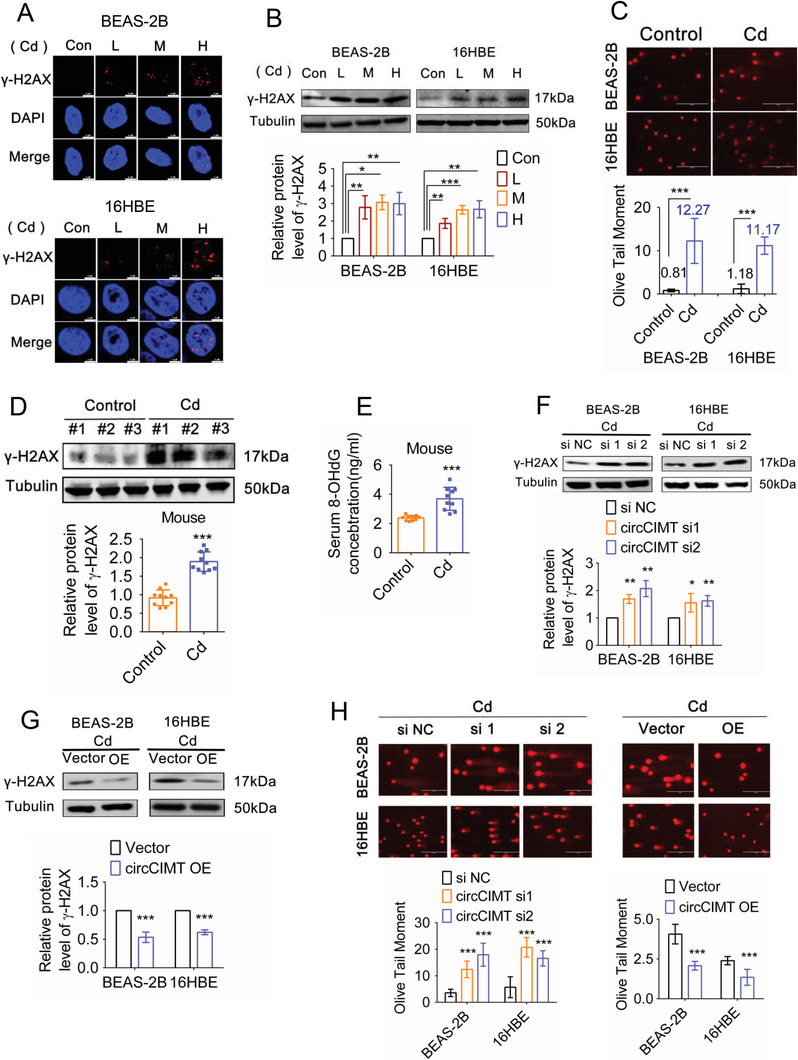
circCIMT suppresses Cd‐induced DNA damage. A) *γ*‐H2AX foci levels were detected in BEAS‐2B and 16HBE cells exposed to different concentrations of Cd by immunofluorescence staining. Scale bar = 5 µM. B) *γ*‐H2AX expression levels were assessed in BEAS‐2B and 16HBE cells exposed to different concentrations of Cd by western blot analysis. C) The comet assay was used to measure DNA damage levels in Cd‐treated BEAS‐2B and 16HBE cells. D) *γ*‐H2AX expression levels were measured in the lung tissue of untreated and Cd‐treated mice by western blotting. n (Control) = 10, n (Cd) = 10. E) Relative 8‐OHdG expression levels were detected in the serum from untreated and Cd‐treated mice. n (Control) = 10, n (Cd) = 10. F,G) The effects of circCIMT knockdown (F) and overexpression (G) on *γ*‐H2AX levels in untreated and Cd‐treated BEAS‐2B and 16HBE cells were examined by western blot analysis. H) The comet assay was used to measure DNA damage levels in Cd‐treated BEAS‐2B and 16HBE cells following circCIMT knockdown and overexpression. *p < 0.05, **p < 0.01, ***p < 0.001.

Gene‐set enrichment analysis (GSEA) revealed that DNA damage response genes were enriched in cells exposed to Cd for 10 weeks (Figure S3A, Supporting Information). The DNA tailing length and the expression level of *γ*‐H2AX together reflect the level of DNA damage in cells. Treatment with grade concentration (L, M, H) of Cd for 48 h led to an increase in *γ*‐H2AX foci, as well as increased *γ*‐H2AX protein expression levels, suggesting that the DNA damage response had been activated by Cd exposure (**Figure** **3**A,B). Our alkaline comet assay showed an increase in Olive Tail Moment (OTM) of DNA following Cd exposure (Figure 3C). In addition, we found that the expression of *γ*‐H2AX in mouse lung tissue and plasma 8‐OHdG (plasma DNA damage marker) levels were also increased after Cd exposure (Figure 3D,E). Thus, taken together, our in vitro and in vivo results suggested that Cd exposure induced DNA damage.

Next, we sought to determine the role of circCIMT in Cd‐induced DNA damage. In untreated cells, knockdown and overexpression of circCIMT did not affect *γ*‐H2AX levels, suggesting that circCIMT does not regulate DNA damage in the absence of Cd (Figure S3B,C, Supporting Information). However, following Cd exposure, increased *γ*‐H2AX expression and nuclear foci were observed in circCIMT‐silenced cells, while overexpression of circCIMT had the opposite effect (Figure 3F,G and Figure S3D,E, Supporting Information). Our alkaline comet assay showed that the OTM was increased in circCIMT‐silenced cells exposed to Cd, but decreased in circCIMT‐overexpressing cells (Figure 3H). We also analyzed the correlation between 8‐OHdG and circCIMT in the mouse blood, and confirmed the negative correlation between circCIMT and DNA damage (Figure S3F, Supporting Information). These results indicated that circCIMT suppresses DNA damage in lung epithelial cells during exposure to Cd.

### 2.4 circCIMT Interacts with APEX1 to Suppress DNA Damage

**Figure 4:**
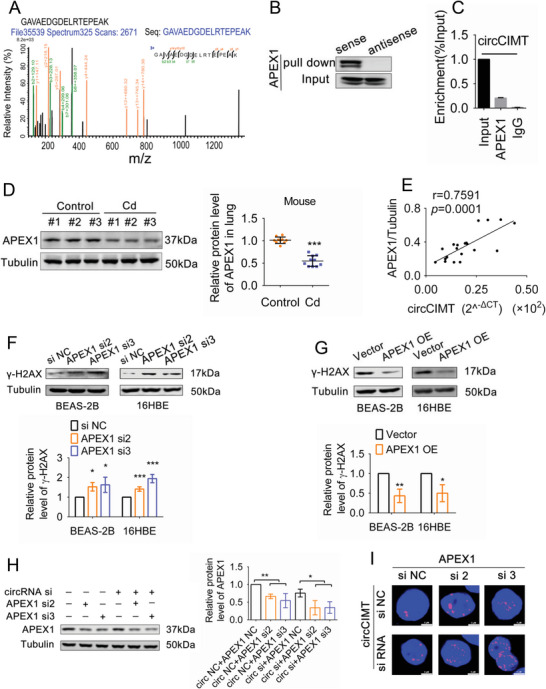
circCIMT interacts with APEX1 to suppress DNA damage. A) Mass spectrometry analysis of APEX1 on the circCIMT RNA pull‐down assay samples. B) The interaction between circCIMT and APEX1 was confirmed by western blot analysis of the circCIMT pull‐down samples. C) The RIP assay using an anti‐APEX1 antibody was used to confirm that circCIMT and APEX1 interacted with each other. D) APEX1 expression in the lung tissue of untreated and Cd‐treated mice was assessed by western blot analysis. n (Control) = 10, n (Cd) = 10. E) Correlation between APEX1 (APEX1/Tubulin) and circCIMT (2^−ΔCT^) in mouse lung tissue. F) *γ*‐H2AX levels in si‐APEX1‐treated BEAS‐2B and 16HBE cells were assessed by western blot analysis. G) *γ*‐H2AX levels in APEX1‐overexpressing BEAS‐2B and 16HBE cells were examined by western blot analysis. H) Western blot analysis of APEX1 protein expression levels in 16HBE cells after simultaneous knockdown of circCIMT and APEX1. I) The number of *γ*‐H2AX foci was detected by immunofluorescence staining after circCIMT and APEX1 co‐interference. Scale bar = 5 µM. *p < 0.05, **p < 0.01, ***p < 0.001.

Next, we examined how circCIMT mediates the DNA damage response. DNA damage repair proteins mediate the DNA damage response and promote gene repair, and are therefore key factors in the maintenance of genomic stability. Here, we constructed biotin‐labeled probes specifically targeting the circCIMT junction site and used a circRNA pull‐down assay to capture proteins directly associated with circCIMT (Figure S4A, Supporting Information). Mass spectrometry revealed that APEX1, a protein involved in DNA damage repair, was enriched in the protein complex obtained by the circCIMT pull down assay (**Figure** **4**A). Western blot analysis further confirmed the presence of APEX1 in the circCIMT pull‐down protein complex solution (Figure 4B). RIP analysis demonstrated that APEX1 and circCIMT interacted with each other (Figure 4C). Subsequently, in order to more accurately detect the binding region of APEX1 on circCIMT, we divided the complete circCIMT sequence into three fragments (circCIMT#1, circCIMT#2, circCIMT#3) and constructed three corresponding probes to implement the pull‐down assay (Figure S4B, Supporting Information). We found significantly higher levels of APEX1 enrichment with the circCIMT#2 probe, containing the circCIMT junction site (Figure S4C, Supporting Information). These results confirmed the binding relationship between circCIMT and APEX1, and demonstrated that binding depended on the unique junction sequence of circCIMT.

Next, we sought to determine whether circCIMT affected DNA damage through APEX1. First, we found that APEX1 was significantly down‐regulated in mouse lung tissue following Cd exposure (Figure 4D). Furthermore, we found that APEX1 expression in mouse lung tissue was positively correlated with circCIMT expression (Figure 4E). After establishing the effectiveness of si‐APEX1 (Figure S4D, Supporting Information), we found that knockdown of APEX1 led to an increase in *γ*‐H2AX expression during Cd exposure, while overexpression of APEX1 had the opposite effect (Figure 4F,G). Simultaneous knockdown of both circCIMT and APEX1 led to a more significant decrease in APEX1 levels than after the knockdown of circCIMT alone (Figure 4H). Immunofluorescence staining revealed that Cd induced more severe DNA damage in cells co‐transfected with si‐circCIMT and si‐APEX1 than in cells treated with si‐circCIMT or si‐APEX1 alone (Figure 4I). Together, our results suggested that APEX1 is a key molecule that is involved in the regulation of DNA damage by circCIMT.

### 2.5 circCIMT‐Interference Inhibits the Expression of Nuclear Base Excision Repair (BER) Complex Proteins after Cd Exposure

**Figure 5:**
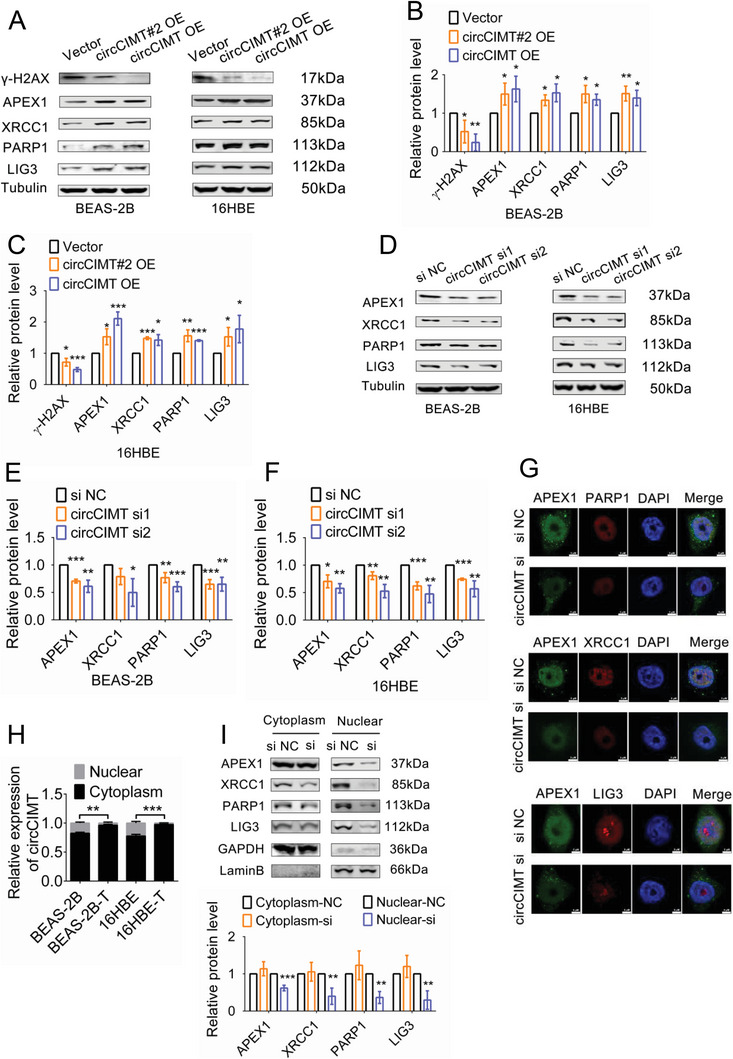
circCIMT‐interference inhibits the expression of nuclear base excision repair (BER) complex proteins after Cd exposure. A–C) Western blot analysis of *γ*‐H2AX, APEX1, XRCC1, PARP1, and LIG3 protein expression levels in Cd‐exposed cells following treatment with circCIMT#2‐ and circCIMT‐overexpression vectors. D–F) Western blot analysis of APEX1, XRCC1, PARP1, and LIG3 protein expression levels in Cd‐treated cells after circCIMT knockdown. G) Co‐localization and expression of APEX1, XRCC1, PARP1, and LIG3 were detected in circCIMT‐silenced cells exposed to Cd by immunofluorescence staining. Scale bar = 5 µM. H) Nuclear/cytoplasmic circCIMT levels were analyzed in control and Cd‐treated cells. I) Western blot analysis of the nuclear/cytoplasmic protein levels of APEX1, XRCC1, PARP1, and LIG3 in Cd‐exposed cells following circCIMT knockdown. *p< 0.05, **p < 0.01, ***p < 0.001.

APEX1 is an endonuclease that participates in the BER pathway by forming complexes with other DNA damage repair proteins, such as XRCC1, PARP1, and LIG3 (Figure S5A, Supporting Information). Thus, we next examined the regulatory relationship between circCIMT and BER proteins during Cd exposure. First, we found that Cd treatment resulted in a decrease in APEX1, XRCC1, PARP1, and LIG3 protein expression levels, indicating that Cd exposure impaired the BER pathway (Figure S5B–D, Supporting Information). During Cd exposure, overexpression of circCIMT#2 or full‐length circCIMT led to a significant increase in the expression of BER complex proteins, together with a decrease in *γ*‐H2AX expression (**Figure** **5**A–C). In addition, we found that knockdown of circCIMT inhibited the expression of BER pathway proteins in lung epithelial cells during Cd treatment (Figure 5D–F). Immunofluorescence staining revealed that APEX1 co‐localized with XRCC1, PARP1, and LIG3, confirming that APEX1 formed protein complexes with these proteins. We also found that knockdown of circCIMT in Cd‐exposed cells significantly reduced APEX1, XRCC1, PARP1, and LIG3 expression levels, which were predominantly expressed in the nucleus (Figure 5G). Next, we extracted nuclear and cytoplasmic RNA fractions from untreated and Cd‐treated cells, and found that in Cd‐exposed cells, circCIMT was significantly reduced in the nucleus, and was predominantly expressed in the cytoplasm (Figure 5H). Next, we separated the cell nuclear and cytoplasmic proteins, and found that circCIMT‐interference led to a significant reduction in BER pathway proteins in the nucleus, but had no significant effect on cytoplasmic levels (Figure 5I). These results suggest that circCIMT‐interference inhibits the DNA damage response by reducing the expression of nuclear BER pathway proteins.

### 2.6 Simultaneous Knockdown of circCIMT and APEX1 Promotes Expression of Tumor‐Associated Genes and Malignant Transformation of Cells

**Figure 6:**
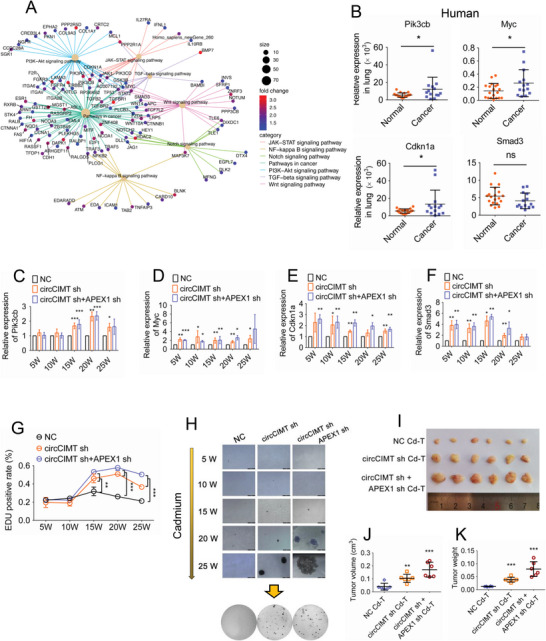
Simultaneous knockdown of circCIMT and APEX1 promotes expression of tumor‐associated genes and malignant transformation of cells. A) KEGG scatter plot of CSC‐like pathway gene sets in si‐circCIMT‐ versus si‐NC‐treated cells. B) Pik3cb, Myc, Cdkn1a, and Smad3 mRNA expression levels in human lung tissue. n (normal) = 19, n (cancer) = 14. C–F) Pik3cb, Myc, Cdkn1a, and Smad3 mRNA expression levels were detected in chronic Cd‐treated cells simultaneously treated with si‐circCIMT and si‐APEX1. G) The EdU assay was used to assess cell proliferation levels in chronic Cd‐exposed cells simultaneously treated with si‐circCIMT and si‐APEX1. H) Anchorage‐independent cell growth ability was measured in 16HBE cells continuously exposed to Cd for 25 weeks. I–K) Tumor volumes and weights in nude mice injected with knockdown of both circCIMT and APEX1 cells and exposed to Cd. n (NC) = 6, n (circCIMT sh) = 6, n (circCIMT sh+APEX1 sh) = 6. *p < 0.05, **p < 0.01, ***p < 0.001

Given that abnormalities in cell signaling pathways are associated with tumor progression, we next investigated the gene transcription profiles following circCIMT interference. GSEA revealed that knockdown of circCIMT in Cd‐exposed cells led to up‐regulation of tumor‐related genes (Figure S6A, Supporting Information), including cellular senescence, FOXO signaling pathway, MAPK signaling pathway, P53 signaling pathway, and transcriptional signaling pathway (Figure S6B, Supporting Information). These findings may explain the growth advantages of circCIMT‐silenced cells after Cd treatment. The KEGG scatter plot revealed that multiple genes intersected with the CSC‐related pathways, including Gsk3b, Notch2, Pik3cb, Myc, Cdkn1a and Smad3, which were the focus of our analysis (**Figure** **6**A and Figure S6C, Supporting Information). We also found that these genes were up‐regulated in Cd‐transformed cells, as well as in mouse lung tissue after long‐term exposure to Cd (Figure S6D–F, Supporting Information). Furthermore, Pik3cb, Myc, Cdkn1a, and Smad3 were found to be negatively correlated with circCIMT (Figure S6G, Supporting Information). In human samples, high expression levels of Pik3cb, Myc, and Cdkn1a were also observed in lung cancer tissue (Figure 6B). We next silenced expression of both circCIMT and APEX1 in cells exposed to Cd for 48 h, and found that Pik3cb, Myc, Cdkn1a, and Smad3 expression levels were higher than those observed in cells treated with si‐circCIMT alone (Figure S6H–K, Supporting Information). These results revealed that CSC‐related pathways were involved in mediating the effects of circCIMT and APEX1 co‐interference cells after Cd exposure.

In order to further demonstrate that circCIMT and APEX1 mediated malignant transformation in Cd‐exposed cells, we simultaneously knocked down circCIMT and APEX1 in cells continuously exposed to Cd. We found that CSC‐related pathway genes were steadily up‐regulated following knockdown of both circCIMT and APEX1 and chronic Cd exposure (Figure 6C–F). Furthermore, levels of cell proliferation were also increased following chronic exposure to Cd (>15 weeks) (Figure 6G). Simultaneous knockdown of both circCIMT and APEX1 resulted in significant unanchored growth at 20 weeks of Cd exposure, suggesting that a shorter time was required for Cd‐induced malignant transformation than in cells treated with si‐circCIMT alone (Figure 6H). As shown in Figure 6I–K, simultaneous knockdown of circCIMT and APEX1 negatively regulated Cd‐induced malignant transformation and cell growth in our xenograft mouse model. Overall, these data suggest that knockdown of both circCIMT and APEX1 in Cd‐exposed cells, not only promoted the expression of tumor‐associated genes, but also significantly shortened the time taken for cells to undergo malignant transformation.

## 3 Discussion

Exposure to carcinogens, such as Cd, results in changes in gene expression, which promote malignant transformation. Here, we found that down‐regulation of circCIMT and DNA damage were associated with the Cd‐induced malignant transformation of the mouse lung and bronchial epithelial cells. Our results contribute to furthering the understanding of the genetic‐epigenetic interactions mediating the development of Cd‐induced malignant lesions in the lung.

Malignant cells can be transformed from human bronchial epithelial cells during long‐term exposure to heavy metals.^[^ ^19^, ^20^ ^]^ Tumors are fundamentally caused by DNA damage to genetic material.^[^ ^21^, ^22^, ^23^ ^]^ Abnormalities in the DNA damage repair pathway promote genomic instability during Cd exposure, and ultimately drive the malignant transformation of lung epithelial cells. Interestingly, DNA damage has been shown to be accompanied by abnormal expression of non‐coding circRNAs.^[^ ^24^, ^25^, ^26^ ^]^ Mechanistically, circRNAs act primarily by binding microRNAs after gene transcription. In addition, circRNA mechanisms may involve protein binding and regulation of protein function.^[^ ^27^ ^]^ In this study, we found that circCIMT and APEX1 interact, and that the APEX1 binding site on circCIMT contains the reverse loop formation region, consistent with other studies.^[^ ^27^, ^28^ ^]^ APEX1 is a glycosylase involved in the identification of DNA damage sites and base repair during BER, a process used to remove small and non‐helix‐distorting base lesions.^[^ ^29^, ^30^ ^]^ The processes of DNA damage and repair are dynamic, but the expression of circRNA is relatively stable, and the regulatory relationship between them is complex and diverse. We found that knockdown of circCIMT alone did not affect the degree of DNA damage. Moreover, DNA damage and repair in Cd‐exposed cells were imbalanced by down‐regulation of circCIMT, thereby indicating that Cd plays a priming role in the regulation of DNA damage and repair by circRNA, thus confirming the importance of environmental and epigenetic interactions for disease occurrence.^[^ ^31^, ^32^, ^33^ ^]^ Our results showed that circCIMT is necessary to maintain DNA repair. We found that, DNA damage repair proteins function in the nucleus, and circCIMT was mainly distributed in the cytoplasm. Interestingly, in circCIMT‐silenced lung epithelial cells, BER complex proteins in the nucleus were mainly affected. Since circRNAs regulate gene/protein subcellular localization and nuclear/cytoplasmic transport in cells,^[^ ^34^, ^35^ ^]^ circRNAs regulate DNA damage across these regions.

Multiple signaling pathways including Wnt, Notch, and Hedgehog have been implicated in promoting malignant transformation of cells.^[^ ^19^, ^36^ ^]^ Given the well‐established roles of these pathways in tumor initiation and progression, the relationship between circCIMT and these pathways should be considered. Here, we found that Gsk3b, Notch2, Pik3cb, Myc, Cdkn1a, and Smad3 were increased in human lung cancer tissue and Cd‐transformed cells, while Pik3cb, Myc, Cdkn1a, and Smad3 were negatively correlated with circCIMT. Furthermore, simultaneous knockdown of circCIMT and APEX1 led to activation of these genes, suggesting that DNA damage associated with BER abnormalities activated the chemically‐induced carcinogenesis process. In our model, down‐regulation of circCIMT and APEX1, inhibited cell survival in the early stages of chronic Cd exposure, which relates to increased pressure on the cell replication machinery caused by aggravated DNA damage.^[^ ^37^, ^38^, ^39^ ^]^ The increased proliferative ability of cells in the later stages of chronic Cd exposure may be attributed to abnormal amplification caused by instability of the cell genome.^[^ ^40^, ^41^, ^42^ ^]^ More importantly, stable down‐regulation of circCIMT and APEX1 decreased the time taken for lung epithelial cells to acquire tumor characteristics following Cd exposure.

In summary, we report epigenetic evidence that circCIMT acts as a regulator of Cd‐induced malignant transformation, and confirm that circCIMT‐interference promotes chemical carcinogenesis by regulating DNA damage. Thus, our findings provide novel insights into the identification of key genetic‐epigenetic‐environmental interactions in lung cancer.

## 4 Experimental Section

### Animals and Respiratory Exposure to Cadmium

Female BALB/c mice 4–5 weeks old, were purchased from Guangdong Medical Laboratory Animal Center. All animal experiments were conducted in accordance with the guidelines of the Guangzhou Medical University Animal Experiment Ethics Committee (GY2020102, GY2022003). For the mouse model, 0.640 mg m⁻^3^ concentration was calculated on the basis of real‐time exposure data in the human body.^[^ ^2^ ^]^ In this study, mice were exposed to Cd via inhalation using a dynamic oral‐nasal inhalation exposure device for 45 weeks (HOPE‐MED 8050, Tianjing, China). An aerosol preparation system was used to provide a breathing space for multiple mice, thus ensuring that each mouse inhaled the same amount of Cd. CdCl_2_ (202908, Merck, Germany) was dissolved in saline, and transferred to an aerosol generator via a liquid flow pump. Mice were then subjected to whole‐body aerosol exposure of CdCl_2_. Control mice were treated with normal saline.

### Cell Culture and Treatments

As described in previous studies, the human bronchial epithelial (HBE), BEAS‐2B and 16HBE cell lines were cultured using standard procedures.^[^ ^43^, ^44^ ^]^ BEAS‐2B cells were cultured in Bronchial Epithelial Cell Medium (BECM) (#3211, ScienCell, USA), and 16HBE cells were cultured in minimum Eagle's medium (MEM) (01‐026‐1ACS, Biological Industries, China) containing 10% fetal bovine serum (FBS; 11011–8611, Sijiqing, China) and 1% penicillin/streptomycin (15140‐122, Bibco, USA).

Cells were exposed to the corresponding concentration of Cd in the culture medium. For 16HBE cells, it was calculated that the minimum exposure concentration would be 10 µM by estimating the accumulating concentration of Cd in the blood of humans exposed to cigarettes for 1 year.^[^ ^2^ ^]^ Since BEAS‐2B cells were less tolerant, a Cd concentration of 1 µM was selected as the minimum exposure. Cells subjected to acute exposure were collected after 48 h. For chronic treatment, cells were treated with low Cd concentrations 24 h after seeding, then after 48 h Cd exposure, the Cd‐containing medium was removed and cells were subcultured. The above Cd exposure was repeated until malignant transformation of cell morphology occurred. Control cells were not treated with Cd and followed the same passage procedure.

### Patients and Samples

Lung surgery was performed at the Department of Thoracic Surgery, The Six Affiliated Hospital of Guangzhou Medical University (Guangzhou, China). Fourteen lung cancer and para‐carcinoma tissues, and 19 normal lung tissues were collected. Written informed consent was obtained from all patients. This study was approved by the Ethics Committee of Guangzhou Medical University (202101001).

### Measurement of Cd Concentration in Biological Samples

Human urine and mouse plasma samples were analyzed by inductively coupled plasma mass spectrometry (ICP‐MS, Agilent 7900, Japan). Briefly, samples (1 mL) were diluted in nitric acid (3 mL), heated in a microwave for 1 h at 180 °C, then the Cd concentration was determined.

### Whole Transcriptome Sequencing

After 10 weeks of chronic exposure to Cd, total RNA was extracted from control and treated cells. The Illumina HiSeqTM 2500 System (Gene Denovo Biotechnology Co., Ltd, Guangzhou, China) was used to analyze the RNA. Raw data were filtered to obtain clean data, and then TopHat (version 2.0.3.12) was used to perform comparison against the reference genome to identify circRNAs. The read counts obtained from the gene expression level analysis were analyzed in DESeq2 software for gene differential expression analysis. The read count was standardized, the hypothesis testing probability of the model was calculated, and multiple hypothesis testing correction was performed. For mRNA, the libraries were sequenced on the Illumina NovaSeq platform to generate 150 bp paired‐end reads. Hisat2 tools was used for mapping with respect to the reference genome. The data from this study have been deposited in NCBI Sequence Read Archive under accession number SRA: SRP415355. The edge R package was used to determine the differential expression of RNA in different samples, and |fold change| ≥ 2 and *p*<0.05 were considered to indicate statistical significance. All filtered circRNA data are shown in Table S1, Supporting Information.

### RNA Interference and Plasmids

The circCIMT overexpression vector used for transient transfection was pcDNA3.1 and the vector used for stable transfection was LV3‐pcDNA3.1 (GFP). siRNA and overexpression sequences of circCIMT and APEX1 were designed and constructed by GenePharma (Shanghai, China). The siRNA sequences are shown in Table S2, Supporting Information. Cells were transfected using Lipofectamine 3000 reagent (Invitrogen, USA) according to the manufacturer's instructions. Lentiviral vectors containing circCIMT siRNA and APEX1 siRNA were constructed by Hanbio Biotechnology Co., Ltd (Shanghai,China). Specific lentivirus vectors were constructed and packaged. Lentiviral‐transfected cells were selected with puromycin.

### RNA Extraction and qRT‐PCR

Total RNA was extracted from cells and tissues using Trizol reagent (Invitrogen, Carlsbad, CA, USA) according to the manufacturer's instructions. The GoScript^TM^ Reverse Transcription System (Promega, Madison, WI, USA) and random primers were used for reverse transcription. GoTap® qPCR Master Mix (Promega) was used for real‐time PCR in a QuantStudio5 Real‐time PCR System (Applied Biosystems, Foster City, CA, USA). All primer sequences used in this study are shown in Table S3, Supporting Information.

### Western Blot Analysis

Cell and tissue lysates were prepared with RIPA lysis buffer (Beyotime Biotechnology, China). Proteins were separated by SDS‐PAGE, then transferred to polyvinylidene difluoride membranes (Merck Millipore, Billerica, MA, USA). After blocking for 1 h with 5% bovine serum albumin (BSA), membranes were incubated at 4 °C with primary antibodies. The following antibodies were used: *γ*‐H2AX (1:1000, ab26350, Abcam), APEX1 (1:1000, ab268072, Abcam), XRCCI (1:1000, ab134056, Abcam), PARP1 (1:1000, ab191217, Abcam), LIG3 (1:1000, ab185815, Abcam), Aldh1a1 (1:1000, ab52492, Abcam), Sox2 (1:1000, 23064, CST), GAPDH (1:5000, 200306, ZenBio), Tubulin (1:5000, 200608, ZenBio), and lamin B1 (1:1000, ab16048, Abcam). After incubation with goat anti‐rabbit or goat anti‐mouse IRDye™ 800CW secondary antibodies for 1 h at room temperature, images were captured using the Odyssey Imaging System (LI‐COR, Lincoln, NE, USA).

### Immunofluorescence Staining and Immunohistochemistry

Samples were fixed with 4% paraformaldehyde, permeated with 1% Triton X‐100 for 10 min at room temperature, then incubated in 5% BSA for 1 h at room temperature. Samples were then incubated with primary antibodies overnight at 4 °C. The following day, samples were incubated with fluorescent‐labeled rabbit or mouse secondary antibodies (567/488). Nuclei were stained with DAPI. Immunofluorescence images were captured by Leica SP8 confocal microscopy (Leica, Germany). For immunohistochemical staining, following incubation with Aldh1a1 and Sox2 primary antibodies, the avidin‐biotin‐immunoperoxidase technique was applied as previously described.^[^ ^45^ ^]^

### RNA Pull‐Down Assay

The Pierce^TM^ Magnetic RNA‐Protein Pull‐Down Kit (Pierce Biotechnology, Rockford, USA) was used to capture proteins bound to circRNAs. The circCIMT probe is shown in Table S4 (Supporting Information). Briefly, biotin‐labeled RNA was added to magnetic beads (50 µL) and incubated for 30 min at room temperature with agitation. Then, the protein lysate was added and samples were incubated for 1 h with rotation. Next, samples were incubated with the elution buffer for 30 min at 37 °C with rotation. The supernatants were subjected to mass spectrometry analysis. Our data are available within this article and its supplementary data files (Dataset S1, Supporting information).

### RNA Immunoprecipitation (RIP) Assay

Magna RIP^TM^ & EZ‐Magna RIP^TM^ Component Boxes (Pierce Biotechnology, Rockford, USA) were used to enrich the RNA bound to the APEX1 protein. APEX1 and IgG antibodies were added to a magnetic bead suspension and incubated at room temperature for 30 min. The cell lysate supernatant (10 µL) was used as the Input group. The cell lysate (100 µL) was mixed with RIP wash buffer, 0.5 m EDTA and RNase Inhibitor, and incubated overnight at 4 °C. The magnetic beads were treated with protease K solution for 30 min, and the supernatant was retained. RNA was extracted from the supernatants.

### Comet Assay

As described previously, the comet assay was used to analyze levels of DNA damage.^[^ ^46^ ^]^ Briefly, the cell suspension was mixed with 0.5% low‐melting point agarose at 37 °C and transferred to slides pre‐coated with 1% agarose. Samples were incubated for 2 h in the dark. After 20 min of unwinding in the electrophoresis solution, electrophoresis was performed at 25 V voltage for 30 min. DNA damage was measured using a fluorescence microscope (Eclipse Ci, Nikon).

### Migration and Invasion Assay

Transwell porous polycarbonate membrane inserts (Corning, USA) were used for migration experiments, and Matrigel‐coated invasion chambers (BD Biocoat, Corning, USA) were used for invasion experiments. Cells were digested with trypsin to prepare cell suspensions, counted, and adjusted to 1×10^5^/mL. Room add containing 20% fetal bovine serum (600 µL) medium, and then in the room to add 200 µL cell suspension, incubation for 48 h. Invading cells attached to the lower surface of the Transwell chamber. Transwell chambers were collected 48 h later, and cells that had adhered to the lower surface were fixed with 4% paraformaldehyde for 10 min, and stained with crystal violet for 20 min. Cells that remained on the upper surface were removed. Images of the stained cells were captured by microscopy (Nikon).

### Anchorage‐Independent Cell Growth (Soft‐Agar Assay)

The soft‐agar assay was used to measure the anchorage‐independent growth ability of cells. Briefly, 6‐well plates were coated with MEM containing 0.6% low‐melting agarose and 10% FBS. Cells (3 × 10^3^) suspended in MEM containing 0.3% low‐melting agarose and 10% FBS were seeded onto the 6‐well plates, and incubated for 15 days at 37 °C in a 5% CO_2_ incubator. Colonies were counted manually under the microscope and photographed.

### Subcutaneous Tumor Model

Female BALB/c nude mice (5‐week‐old) were used in this experiment. Cells exposed to chronic low‐doses of Cd during circCIMT interference, circCIMT‐APEX1 co‐interference and negative control cells, were suspended in 0.1 mL PBS and inoculated subcutaneously into the flank of nude mice. Three weeks after inoculation, the mice were humanely killed in accordance with the humane treatment policy for animals carrying tumors, and the size of the tumor was observed and measured. All experiments were carried out in accordance with the guidelines of the Guangzhou Medical University Animal Experiment Ethics Committee.

### Statistical Analysis

Statistical analyses were performed by SPSS 22.0 (IBM, Chicago, USA) and visualized by GraphPad Prism (San Diego, USA). Data were presented as average ± SD from at least three independent experiments on cells. Human and mouse data are presented as average ± SD from each group. Student's t test (two‐sided) was used to compare the two groups, and q‐PCR, relative protein quantification, tumor weight and growth, and blood Cd concentration were statistically analyzed. Multiple group comparisons were analyzed by one‐way ANOVA. Correlation analysis and regression analysis were used to analyze the relationships among genes. *p < 0.05, **p < 0.01, ***p < 0.001. *p* values < 0.05 were considered to be statistically significant.

## Conflict of Interest

The authors declare no conflict of interest.

## Author Contributions

M.L., Y.J., and L.H. initiated and designed the study. M.L., W.C., Y.L., and H.Z. conducted the experiments. M.L., W.C., J.C., Q.L., X.Q., and Y.Z. performed animal and patient data analysis. J.C., X.L.,T.L., and Y.J. provided administrative, technical, or material support. M.L., Y.J., and L.H. completed manuscript writing, review, and/or revision. Q.H., Y.L., X.Q., and Y.J. performed study supervision. Y.J. and L.H. provided funding acquisition.

## Supporting information

Supporting Information

Supporting Information Table 1

## Data Availability

The data that support the findings of this study are available from the corresponding author upon reasonable request.
