# Supplementary material for: circCIMT Silencing Promotes Cadmium‐Induced Malignant Transformation of Lung Epithelial Cells Through the DNA Base Excision Repair Pathway
Source: Adv Sci (Weinh). 2023 Feb 22;10(14):2206896. doi: 10.1002/advs.202206896 (PMC10190647; doi:10.1002/advs.202206896)
Supplement: Supplementary file 1 — Supporting Information [file ADVS-10-2206896-s001.pdf]

ADVANCED SCIENCE
Open Access

Supporting Information

for *Adv. Sci.*, DOI 10.1002/advs.202206896

circCIMT Silencing Promotes Cadmium-Induced Malignant Transformation of Lung Epithelial Cells Through the DNA Base Excision Repair Pathway

Meizhen Li, Wei Chen, Jinjin Cui, Qiuyi Lin, Yufei Liu, Huixian Zeng, Qiuhan Hua, Yihui Ling, Xiaodi Qin, Yindai Zhang, Xueqi Li, Tianshu Lin, Lihua Huang and Yiguo Jiang**

Supporting information

circCIMT silencing promotes cadmium-induced malignant transformation of lung epithelial cells through the DNA base excision repair pathway

Meizhen Li, Wei Chen, Jinjin Cui, Qiuyi Lin, Yufei Liu, Huixian Zeng, Qiuhan Hua, Yihui Ling, Xiaodi Qin, Yindai Zhang, Xueqi Li, Tianshu Lin, Lihua Huang, Yiguo Jiang***

Supplementary Material

This supporting information includes:

Figure S1:

Cadmium exposure induces malignant lesions in the mouse lung and malignant transformation of normal bronchial epithelial cells.

Figure S2:

Expression and function of circCIMT in the Cd-induced malignant transformation model.

Figure S3:

circCIMT suppresses Cd-induced DNA damage.

Figure S4:

circCIMT interacts with APEX1 to suppress DNA damage.

Figure S5:

circCIMT-interference inhibits expression of nuclear BER complex proteins after Cd exposure.

Figure S6:

Simultaneous knockdown of circCIMT and APEX1 promotes expression of tumor-associated genes and malignant transformation of cells.

Table S1:

High-throughput circRNA data.

Table S2:

Sequences of oligonucleotides used in this study.

Table S3:

Primers used for quantitative qRT-PCR.

Table S4:

Probes of circCIMT.

Dataset S1:

Proteins of circCIMT pull down.

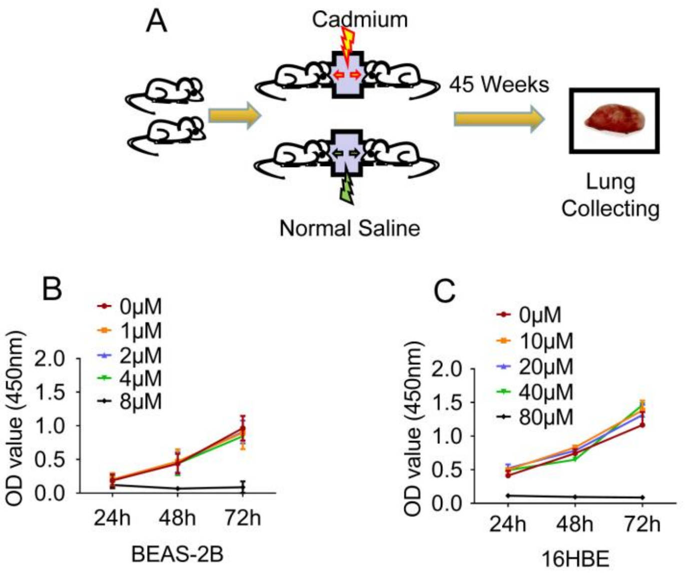

Figure S1. Cadmium exposure induces malignant lesions in the mouse lung and malignant transformation of bronchial epithelial cells. A. Schematic diagram of mouse exposure model. Mice were subjected to chronic Cd exposure via nose and mouth inhalation. Lung tissues were collected after 45 weeks for analysis. **B**, **C**. Relative cell viability levels of BEAS-2B (B) and 16HBE cells (C) exposed to different concentrations of Cd were assessed using the CCK8 assay.

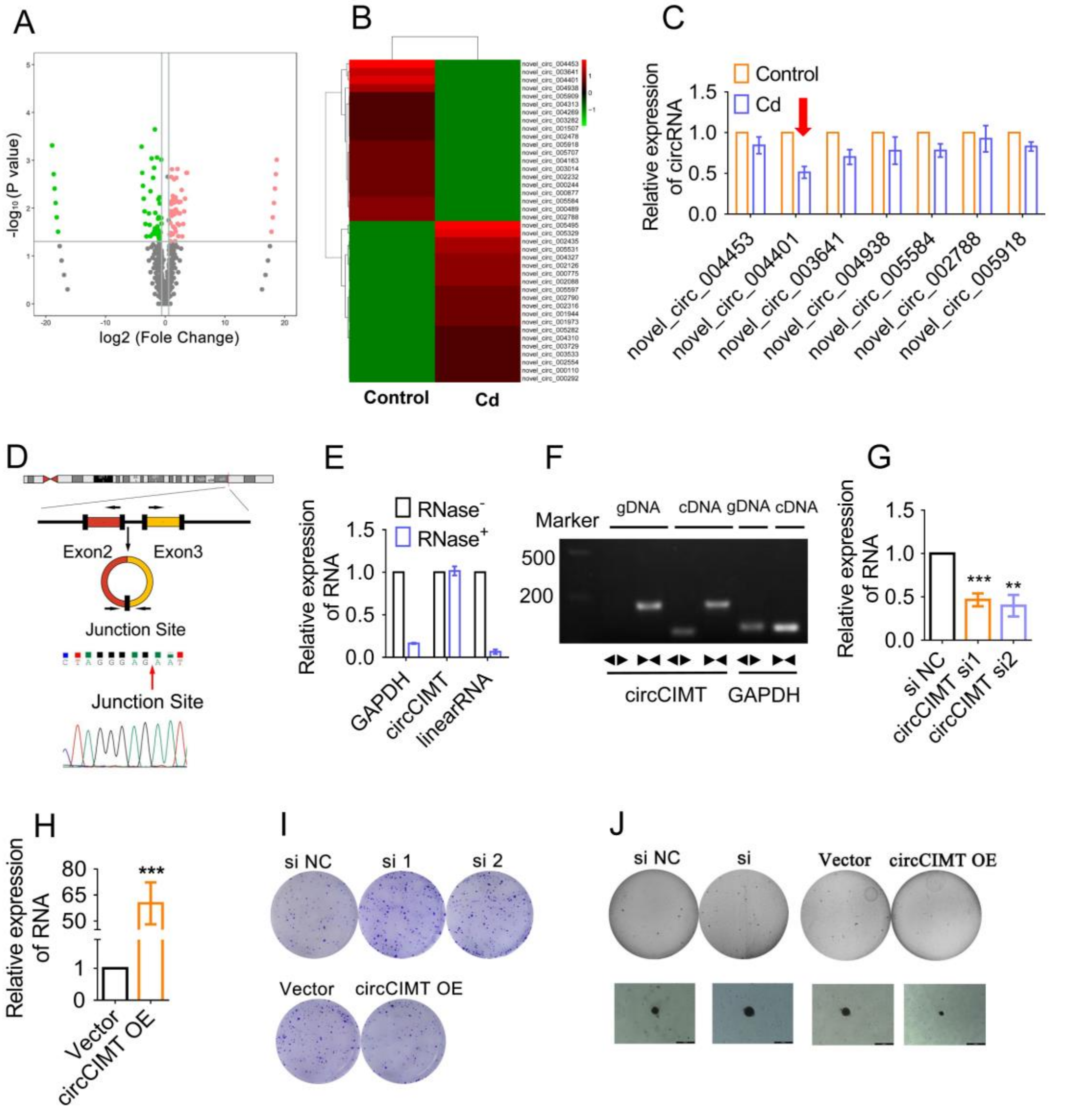

Figure S2. Expression and function of circCIMT in a Cd-induced malignant transformation model. A. Volcano plot was used to compare the differential expression of circRNAs in untreated and Cd-treated cells. The red dots represent up-regulated circRNAs and the green dots represent down-regulated circRNAs in Cd-treated cells with $p < 0.05$. **B**. Heat map of 20 up-regulated and 20 down-regulated circRNAs in Cd-treated cells compared to control cells ($p < 0.05$). **C**. The top 7 down-regulated circRNAs in Cd-treated cells were detected by q-PCR. **D**. Schematic representation of circCIMT. circCIMT is derived from exon 2 and exon 3 of the EZH2 gene. The back-splice junction of circCIMT was identified by Sanger sequencing. **E**. The expression of circCIMT and linear mRNA were detected after treatment with RNase R. **F**. Convergent and divergent primers were used to analyze circCIMT and linear mRNA levels in cDNA and genomic DNA (gDNA). **G. H.** The interference (**G**) and

overexpression (**H**) efficiency of circCIMT were detected by q-PCR. **I**. The colony formation ability was assessed in Cd-treated cells undergoing malignant transformation after circCIMT knockdown and overexpression. **J**. Anchorage-independent growth ability was examined in Cd-treated cells undergoing malignant transformation after circCIMT knockdown and overexpression.

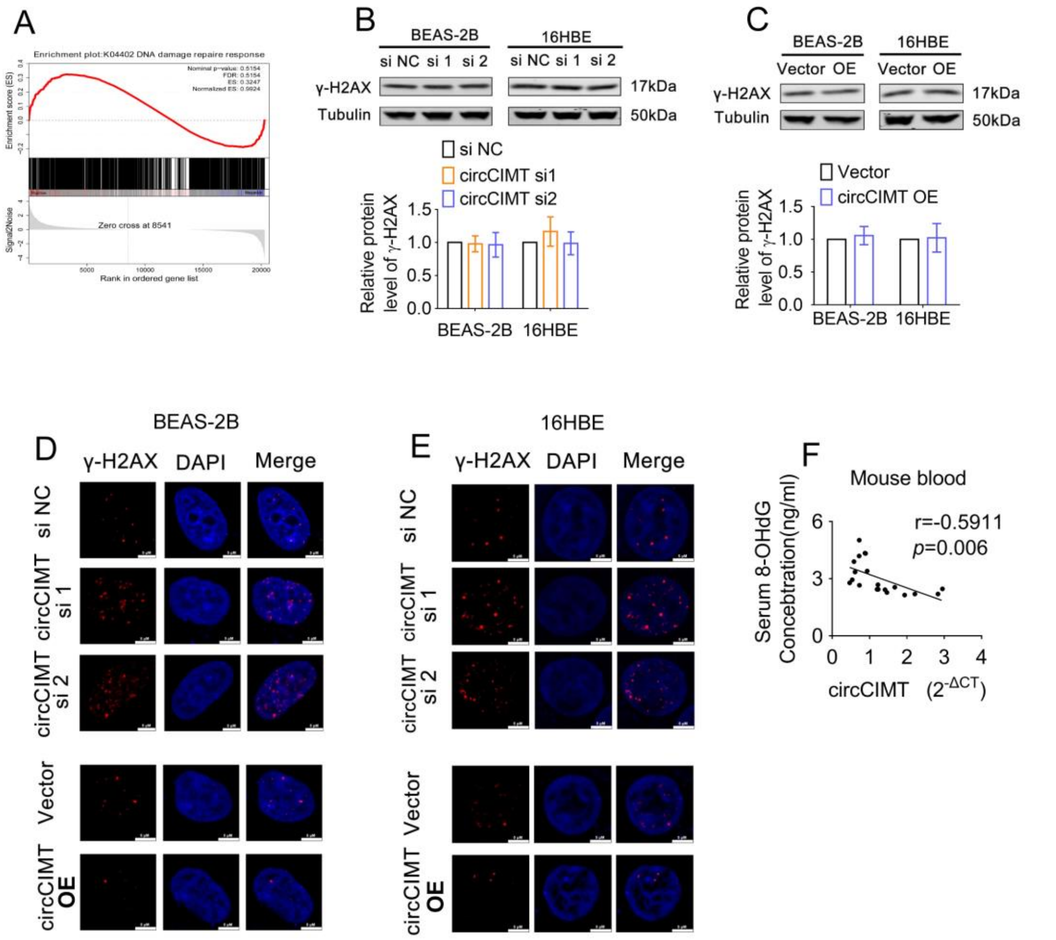

Figure S3. circCIMT suppresses Cd-induced DNA damage. A. Gene set enrichment analysis (GSEA) showing the relative enrichment of DNA damage repair response genes in the Cd-treated group compared to untreated cells. **B**. Western blot analysis of γ-H2AX levels in BEAS-2B and 16HBE cells following si-circCIMT treatment. **C**. Western blot analysis of γ-H2AX levels in BEAS-2B and 16HBE cells following circCIMT overexpression. **D**, **E**. Immunofluorescence staining showing the number of γ-H2AX foci in Cd-exposed BEAS-2B (**D**) and 16HBE cells (**E**) following circCIMT knockdown and overexpression. Scale bar = 5 μM. **F**. Correlation between 8-OHdG (ng/ml) and circCIMT ($2^{-\Delta CT}$) in mouse blood. n = 20.

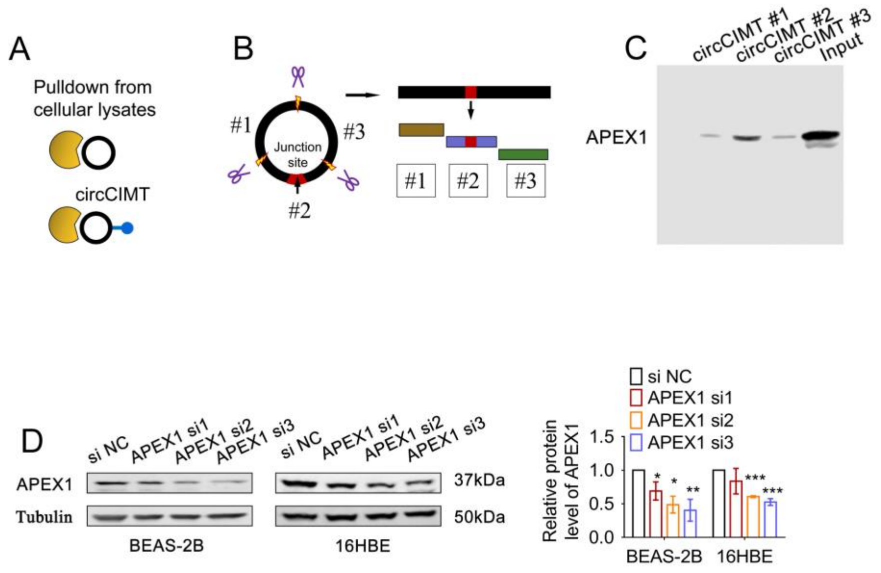

Figure S4. circCIMT interacts with APEX1 to suppress DNA damage. A. Schematic diagram showing the circRNA pull-down assay. **B**. Schematic representation of three circCIMT fragments (circCIMT#1, circCIMT#2, circCIMT#3) obtained by dividing the complete circCIMT. **C**. Western blot analysis was used to detect levels of APEX1 enrichment in the pull-down samples obtained from the three circCIMT fragments. **D**. Western blot analysis of APEX1 expression levels in BEAS-2B and 16HBE cells after APEX1 interference. **p* < 0.05, ***p* < 0.01, ****p* < 0.001.

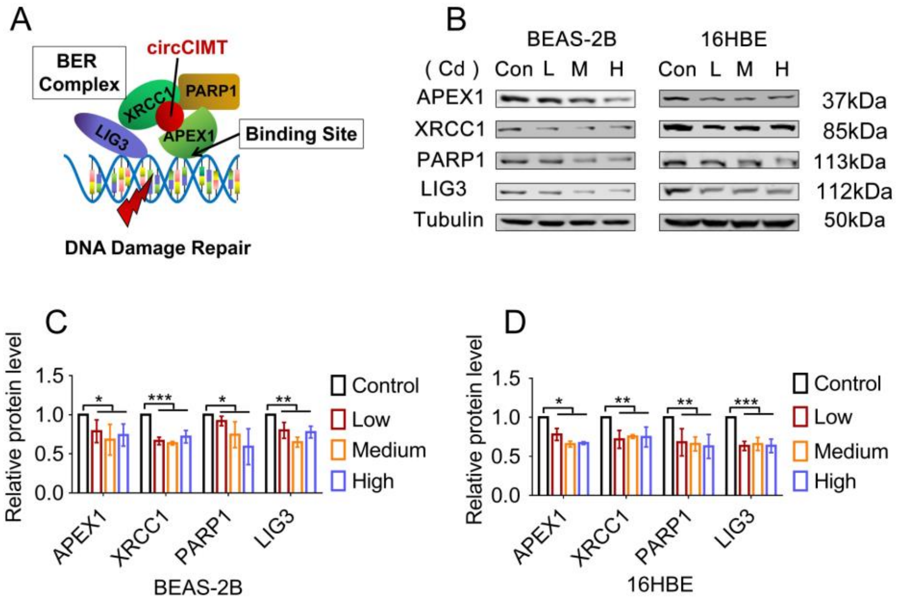

Figure S5. circCIMT-interference inhibits expression of nuclear BER complex proteins after Cd exposure. A. Schematic illustration of circCIMT binding to the BER protein complex, which is involved in the DNA damage repair process. **B-D**. Western blot analysis of APEX1, XRCC1, PARP1 and LIG3 protein expression levels in Cd-treated bronchial epithelial cells. *$p < 0.05$, **$p < 0.01$, ***$p < 0.001$.

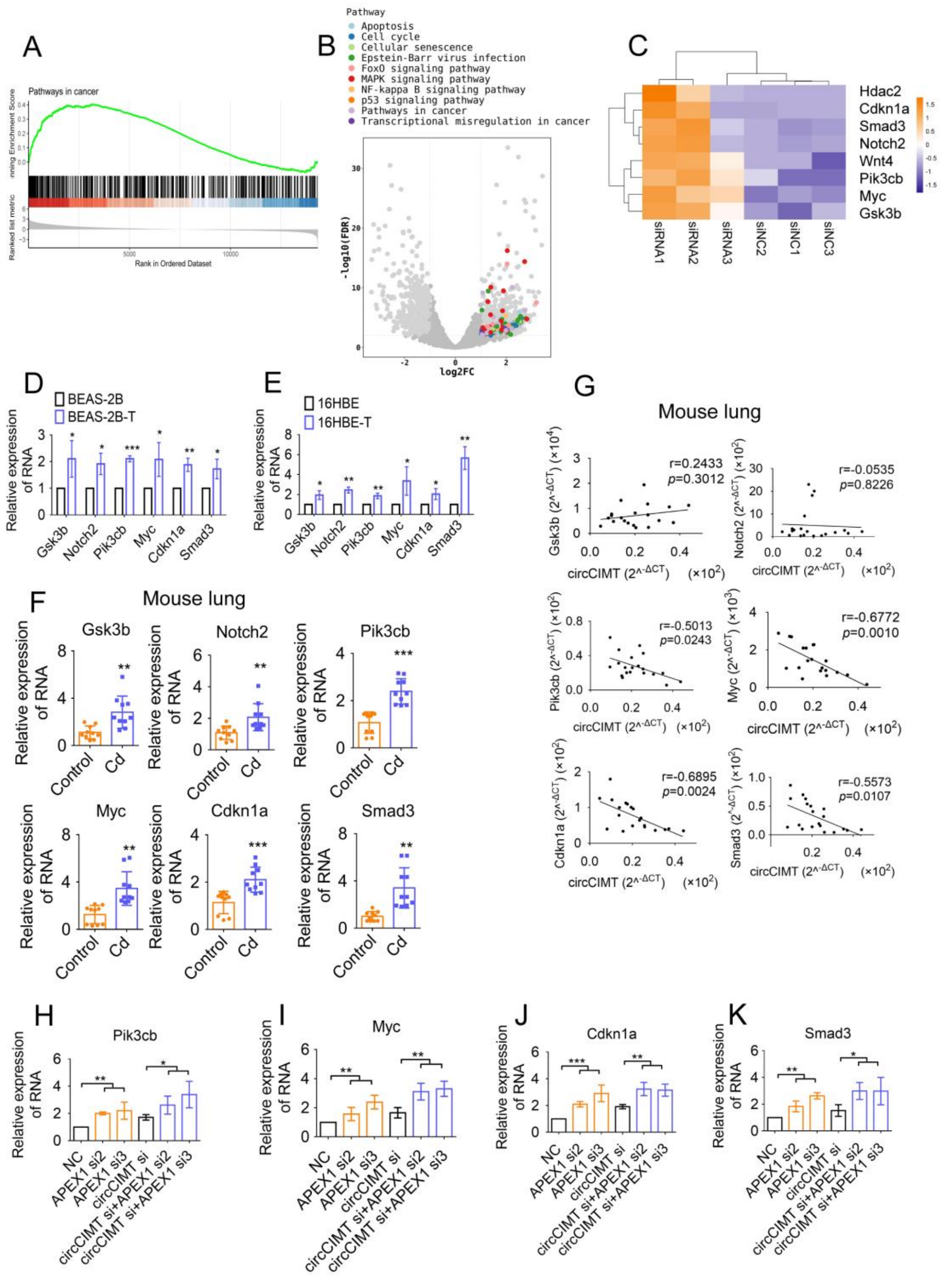

Figure S6. Simultaneous knockdown of circCIMT and APEX1 promotes expression of tumor-associated genes and malignant transformation of cells. A. Gene set enrichment analysis showing relative enrichment of tumor-related genes in circCIMT-silenced cells. **B**. Volcano plot of abnormally expressed genes in si-circCIMT versus si-NC cells. Genes associated with the cell cycle, FOXO signaling pathway, MAPK signaling pathway, P53 signaling pathway, and transcriptional signaling pathways were up-regulated in

circCIMT-silenced cells during early malignant transformation. **C**. Heat map of the CSC-like pathway key genes in circCIMT-silenced cells compared to si-NC-treated cells. **D**. mRNA expression of Gsk3b, Notch2, Pik3cb, Myc, Cdkn1a, and Smad3 in BEAS-2B and BEAS-2B-T cells. **E**. mRNA expression of Gsk3b, Notch2, Pik3cb, Myc, Cdkn1a, and Smad3 in 16HBE and 16HBE-T cells. **F**. q-PCR analysis of Gsk3b, Notch, Pik3cb, Myc, Cdkn1a, and Smad3 mRNA levels in mice continuously exposed to Cd. n (Control) = 10, n (Cd) = 10. **G**. Correlation between circCIMT, and Gsk3b, Notch2, Pik3cb, Myc, Cdkn1a, and Smad3 ($2^{-\Delta CT}$) in mouse lung tissue. n = 20. **H**-**K**. Effects of simultaneous knockdown of circCIMT and APEX1 on Pik3cb, Myc, Cdkn1a, and Smad3 mRNA levels after chronic exposure to Cd. *$p < 0.05$, **$p < 0.01$, ***$p < 0.001$.

Table S1. High-throughput circRNA data.

ID	Control_ count	Cd_ count	log2(FC)	*P* Value	circRNA
novel_circ_000015	20	45	1.0291653	0.008247832	hsa_circ_0006471
novel_circ_000106	0	6	17.801791	0.03127344	hsa_circ_0055927
novel_circ_000110	1	8	2.859240299	0.039104693	novel
novel_circ_000191	2	15	2.766130894	0.00419502	hsa_circ_0036963
novel_circ_000211	8	0	-18.3575882	0.007823441	hsa_circ_000831
novel_circ_000219	24	12	-1.140759701	0.028958901	hsa_circ_0006127
novel_circ_000231	0	7	18.02418342	0.01564141	hsa_circ_0067087
novel_circ_000233	0	6	17.801791	0.03127344	hsa_circ_000479
novel_circ_000244	9	1	-3.310684703	0.021515144	hsa_circ_0083383
novel_circ_000286	8	1	-3.140759701	0.039104693	hsa_circ_0005781
novel_circ_000292	1	8	2.859240299	0.039104693	hsa_circ_0006971
novel_circ_000295	10	0	-18.67951629	0.001957522	hsa_circ_0090434
novel_circ_000311	0	8	18.2168285	0.007823441	hsa_circ_0007907
novel_circ_000326	0	6	17.801791	0.03127344	hsa_circ_0066784
novel_circ_000328	0	8	18.2168285	0.007823441	novel
novel_circ_000337	0	11	18.67626012	0.00097925	hsa_circ_0005564
novel_circ_000340	0	6	17.801791	0.03127344	hsa_circ_0044793
novel_circ_000357	2	12	2.444202799	0.012969461	hsa_circ_0047721
novel_circ_000384	2	10	2.181168394	0.038630625	hsa_circ_000536
novel_circ_000414	0	8	18.2168285	0.007823441	novel
novel_circ_000473	0	8	18.2168285	0.007823441	novel
novel_circ_000489	10	1	-3.462687796	0.011740243	hsa_circ_0003651
novel_circ_000492	36	12	-1.725722202	0.00022598	hsa_circ_001894
novel_circ_000495	0	10	18.53875659	0.001957522	hsa_circ_0005725
novel_circ_000518	9	0	-18.5275132	0.003913285	novel
novel_circ_000582	7	0	-18.16494312	0.01564141	hsa_circ_0072714
novel_circ_000664	6	0	-17.9425507	0.03127344	novel
novel_circ_000680	0	7	18.02418342	0.01564141	hsa_circ_0044839
novel_circ_000700	6	0	-17.9425507	0.03127344	novel
novel_circ_000707	6	0	-17.9425507	0.03127344	novel
novel_circ_000741	0	9	18.3867535	0.003913285	novel
novel_circ_000750	9	24	1.274277798	0.035230821	hsa_circ_0007714
novel_circ_000775	1	10	3.181168394	0.011740243	hsa_circ_0078201
novel_circ_000801	0	7	18.02418342	0.01564141	hsa_circ_0033130
novel_circ_000809	6	0	-17.9425507	0.03127344	novel
novel_circ_000833	0	6	17.801791	0.03127344	hsa_circ_0078200

novel_circ_000837	2	13	2.559680017	0.007406097	novel
novel_circ_000877	9	1	-3.310684703	0.021515144	hsa_circ_0004366
novel_circ_000912	6	0	-17.9425507	0.03127344	hsa_circ_0003045
novel_circ_000929	8	0	-18.3575882	0.007823441	hsa_circ_0081006
novel_circ_000949	0	10	18.53875659	0.001957522	hsa_circ_0018038
novel_circ_000981	5	23	2.06087416	0.001524329	novel
novel_circ_001004	0	6	17.801791	0.03127344	hsa_circ_0072758
novel_circ_001025	5	17	1.624775045	0.026681361	hsa_circ_0018992
novel_circ_001032	27	13	-1.195207485	0.01669585	hsa_circ_0005455
novel_circ_001061	0	6	17.801791	0.03127344	hsa_circ_0006323
novel_circ_001092	5	19	1.785239717	0.010665387	hsa_circ_0005035
novel_circ_001119	0	7	18.02418342	0.01564141	hsa_circ_0081854
novel_circ_001131	0	6	17.801791	0.03127344	novel
novel_circ_001133	2	13	2.559680017	0.007406097	novel
novel_circ_001149	23	12	-1.079359157	0.041134536	hsa_circ_001035
novel_circ_001198	21	10	-1.211149029	0.029573372	novel
novel_circ_001247	0	9	18.3867535	0.003913285	hsa_circ_0047022
novel_circ_001277	0	6	17.801791	0.03127344	novel
novel_circ_001297	0	6	17.801791	0.03127344	hsa_circ_0007159
novel_circ_001313	0	8	18.2168285	0.007823441	novel
novel_circ_001339	2	12	2.444202799	0.012969461	hsa_circ_0007915
novel_circ_001362	12	3	-2.140759701	0.035222917	hsa_circ_0008011
novel_circ_001367	10	2	-2.462687796	0.038630625	hsa_circ_0007368
novel_circ_001380	0	6	17.801791	0.03127344	novel
novel_circ_001465	2	10	2.181168394	0.038630625	hsa_circ_0008383
novel_circ_001507	8	1	-3.140759701	0.039104693	hsa_circ_0006780
novel_circ_001525	6	0	-17.9425507	0.03127344	novel
novel_circ_001549	6	0	-17.9425507	0.03127344	hsa_circ_0008272
novel_circ_001556	6	0	-17.9425507	0.03127344	novel
novel_circ_001578	13	35	1.288083598	0.0055707	novel
novel_circ_001652	8	25	1.503096489	0.013606508	hsa_circ_0003979
novel_circ_001656	0	7	18.02418342	0.01564141	hsa_circ_001879
novel_circ_001677	6	0	-17.9425507	0.03127344	hsa_circ_002025
novel_circ_001692	0	6	17.801791	0.03127344	hsa_circ_0004623
novel_circ_001702	7	0	-18.16494312	0.01564141	hsa_circ_0008471
novel_circ_001713	8	0	-18.3575882	0.007823441	hsa_circ_0029620
novel_circ_001753	6	0	-17.9425507	0.03127344	hsa_circ_0091669
novel_circ_001754	6	0	-17.9425507	0.03127344	hsa_circ_0007615
novel_circ_001760	10	2	-2.462687796	0.038630625	hsa_circ_0002025
novel_circ_001815	7	0	-18.16494312	0.01564141	hsa_circ_0076198

novel_circ_001816	7	0	-18.16494312	0.01564141	novel
novel_circ_001852	0	8	18.2168285	0.007823441	hsa_circ_0054876
novel_circ_001865	0	6	17.801791	0.03127344	hsa_circ_0080035
novel_circ_001879	0	6	17.801791	0.03127344	hsa_circ_0005859
novel_circ_001922	0	9	18.3867535	0.003913285	novel
novel_circ_001924	0	9	18.3867535	0.003913285	hsa_circ_0002563
novel_circ_001928	6	0	-17.9425507	0.03127344	hsa_circ_0003431
novel_circ_001944	1	9	3.0291653	0.021515144	hsa_circ_0071174
novel_circ_001961	6	0	-17.9425507	0.03127344	hsa_circ_001731
novel_circ_001970	6	0	-17.9425507	0.03127344	novel
novel_circ_001973	1	9	3.0291653	0.021515144	hsa_circ_0049434
novel_circ_002062	2	12	2.444202799	0.012969461	novel
novel_circ_002088	1	10	3.181168394	0.011740243	novel
novel_circ_002098	0	6	17.801791	0.03127344	novel
novel_circ_002104	0	8	18.2168285	0.007823441	novel
novel_circ_002126	1	10	3.181168394	0.011740243	hsa_circ_0085441
novel_circ_002140	0	8	18.2168285	0.007823441	novel
novel_circ_002160	4	14	1.666595221	0.049136186	hsa_circ_0004405
novel_circ_002168	0	6	17.801791	0.03127344	hsa_circ_0079492
novel_circ_002180	0	6	17.801791	0.03127344	hsa_circ_0008171
novel_circ_002185	11	34	1.487271521	0.002485965	hsa_circ_001517
novel_circ_002201	11	30	1.306699276	0.011591261	hsa_circ_002036
novel_circ_002215	2	10	2.181168394	0.038630625	hsa_circ_0008599
novel_circ_002232	9	1	-3.310684703	0.021515144	hsa_circ_002094
novel_circ_002244	0	6	17.801791	0.03127344	hsa_circ_0059581
novel_circ_002245	6	0	-17.9425507	0.03127344	novel
novel_circ_002247	6	0	-17.9425507	0.03127344	hsa_circ_0038487
novel_circ_002266	12	3	-2.140759701	0.035222917	hsa_circ_0067682
novel_circ_002284	36	18	-1.140759701	0.006527998	hsa_circ_0046999
novel_circ_002316	1	9	3.0291653	0.021515144	hsa_circ_000467
novel_circ_002324	8	0	-18.3575882	0.007823441	novel
novel_circ_002379	6	0	-17.9425507	0.03127344	hsa_circ_0089254
novel_circ_002381	0	6	17.801791	0.03127344	hsa_circ_0047125
novel_circ_002435	1	11	3.318671917	0.006362167	hsa_circ_001256
novel_circ_002440	20	9	-1.292762795	0.024220351	hsa_circ_000524
novel_circ_002460	6	0	-17.9425507	0.03127344	novel
novel_circ_002478	8	1	-3.140759701	0.039104693	hsa_circ_0005406
novel_circ_002514	4	17	1.94670314	0.011858624	hsa_circ_0085458
novel_circ_002532	22	53	1.127729135	0.001546482	hsa_circ_001662
novel_circ_002542	8	0	-18.3575882	0.007823441	novel

novel_circ_002554	1	8	2.859240299	0.039104693	hsa_circ_0008618
novel_circ_002688	0	6	17.801791	0.03127344	hsa_circ_0017787
novel_circ_002706	40	17	-1.375224955	0.000876429	hsa_circ_0005729
novel_circ_002729	0	6	17.801791	0.03127344	novel
novel_circ_002762	6	0	-17.9425507	0.03127344	hsa_circ_0070476
novel_circ_002782	6	0	-17.9425507	0.03127344	hsa_circ_0006855
novel_circ_002788	10	1	-3.462687796	0.011740243	hsa_circ_0015928
novel_circ_002790	1	9	3.0291653	0.021515144	hsa_circ_0004431
novel_circ_002795	6	0	-17.9425507	0.03127344	hsa_circ_001434
novel_circ_002826	0	7	18.02418342	0.01564141	novel
novel_circ_002917	4	14	1.666595221	0.049136186	hsa_circ_0008549
novel_circ_002944	7	0	-18.16494312	0.01564141	hsa_circ_0002724
novel_circ_003014	9	1	-3.310684703	0.021515144	hsa_circ_0009029
novel_circ_003017	23	7	-1.856966735	0.001442216	hsa_circ_001767
novel_circ_003091	6	0	-17.9425507	0.03127344	hsa_circ_0004899
novel_circ_003110	0	6	17.801791	0.03127344	novel
novel_circ_003114	22	5	-2.278263225	0.00091941	hsa_circ_0008567
novel_circ_003118	7	0	-18.16494312	0.01564141	novel
novel_circ_003163	6	0	-17.9425507	0.03127344	hsa_circ_0089722
novel_circ_003166	10	0	-18.67951629	0.001957522	hsa_circ_0008755
novel_circ_003183	0	6	17.801791	0.03127344	hsa_circ_0004940
novel_circ_003231	6	0	-17.9425507	0.03127344	novel
novel_circ_003251	6	0	-17.9425507	0.03127344	hsa_circ_0029625
novel_circ_003258	6	0	-17.9425507	0.03127344	hsa_circ_0003898
novel_circ_003274	0	7	18.02418342	0.01564141	hsa_circ_0037517
novel_circ_003282	8	1	-3.140759701	0.039104693	hsa_circ_001027
novel_circ_003288	0	6	17.801791	0.03127344	novel
novel_circ_003307	0	6	17.801791	0.03127344	novel
novel_circ_003340	0	10	18.53875659	0.001957522	novel
novel_circ_003364	0	6	17.801791	0.03127344	novel
novel_circ_003391	7	0	-18.16494312	0.01564141	hsa_circ_0008737
novel_circ_003404	4	14	1.666595221	0.049136186	novel
novel_circ_003410	15	5	-1.725722202	0.026681361	hsa_circ_000026*
novel_circ_003461	9	0	-18.5275132	0.003913285	hsa_circ_0002448
novel_circ_003467	0	7	18.02418342	0.01564141	novel
novel_circ_003478	10	2	-2.462687796	0.038630625	hsa_circ_0003192
novel_circ_003482	7	0	-18.16494312	0.01564141	hsa_circ_0002654
novel_circ_003533	1	8	2.859240299	0.039104693	novel
novel_circ_003540	7	0	-18.16494312	0.01564141	novel
novel_circ_003545	0	6	17.801791	0.03127344	hsa_circ_0056189

novel_circ_003562	0	8	18.2168285	0.007823441	hsa_circ_0022383
novel_circ_003564	15	3	-2.462687796	0.004442811	novel
novel_circ_003623	24	12	-1.140759701	0.028958901	hsa_circ_000153
novel_circ_003625	11	2	-2.60019132	0.022502846	hsa_circ_0007723
novel_circ_003641	12	1	-3.725722202	0.003427498	hsa_circ_0009000
novel_circ_003643	0	8	18.2168285	0.007823441	novel
novel_circ_003670	9	28	1.496670219	0.007686922	hsa_circ_0006332
novel_circ_003710	0	6	17.801791	0.03127344	hsa_circ_0063870
novel_circ_003729	1	8	2.859240299	0.039104693	hsa_circ_0029775
novel_circ_003730	2	10	2.181168394	0.038630625	hsa_circ_0005221
novel_circ_003742	6	24	1.859240299	0.002332015	hsa_circ_0084188
novel_circ_003748	12	33	1.318671917	0.006668724	hsa_circ_0004630
novel_circ_003759	3	14	2.08163272	0.021320761	hsa_circ_001932
novel_circ_003783	0	6	17.801791	0.03127344	hsa_circ_0031841
novel_circ_003841	6	0	-17.9425507	0.03127344	novel
novel_circ_003861	6	0	-17.9425507	0.03127344	novel
novel_circ_003970	7	0	-18.16494312	0.01564141	hsa_circ_0043954
novel_circ_003979	2	11	2.318671917	0.022502846	hsa_circ_0071616
novel_circ_004074	6	0	-17.9425507	0.03127344	novel
novel_circ_004076	0	9	18.3867535	0.003913285	novel
novel_circ_004086	13	4	-1.841199419	0.030957355	hsa_circ_001303
novel_circ_004135	0	8	18.2168285	0.007823441	hsa_circ_0004945
novel_circ_004163	9	1	-3.310684703	0.021515144	hsa_circ_0017412
novel_circ_004192	0	6	17.801791	0.03127344	novel
novel_circ_004201	12	3	-2.140759701	0.035222917	hsa_circ_0032008
novel_circ_004214	11	2	-2.60019132	0.022502846	hsa_circ_0009038
novel_circ_004215	0	7	18.02418342	0.01564141	novel
novel_circ_004218	6	0	-17.9425507	0.03127344	novel
novel_circ_004265	6	0	-17.9425507	0.03127344	hsa_circ_0005507
novel_circ_004269	8	1	-3.140759701	0.039104693	hsa_circ_0003815
novel_circ_004303	6	0	-17.9425507	0.03127344	hsa_circ_0011889
novel_circ_004310	1	8	2.859240299	0.039104693	hsa_circ_0004179
novel_circ_004313	8	1	-3.140759701	0.039104693	hsa_circ_0003168
novel_circ_004327	1	10	3.181168394	0.011740243	hsa_circ_0004069
novel_circ_004334	6	0	-17.9425507	0.03127344	novel
novel_circ_004375	0	8	18.2168285	0.007823441	hsa_circ_0010109
novel_circ_004401	13	1	-3.841199419	0.00183717	hsa_circ_0006357
novel_circ_004412	14	31	1.006081687	0.035903799	hsa_circ_0061395
novel_circ_004419	16	6	-1.555797201	0.034791993	hsa_circ_0008197
novel_circ_004443	4	14	1.666595221	0.049136186	hsa_circ_0060762

novel_circ_004453	14	1	-3.948114623	0.000521183	hsa_circ_001174
novel_circ_004489	6	0	-17.9425507	0.03127344	hsa_circ_0003698
novel_circ_004528	0	6	17.801791	0.03127344	novel
novel_circ_004533	6	0	-17.9425507	0.03127344	hsa_circ_0036146
novel_circ_004536	0	6	17.801791	0.03127344	hsa_circ_0087036
novel_circ_004564	6	0	-17.9425507	0.03127344	novel
novel_circ_004566	2	13	2.559680017	0.007406097	hsa_circ_0075158
novel_circ_004616	6	0	-17.9425507	0.03127344	hsa_circ_0003490
novel_circ_004649	8	0	-18.3575882	0.007823441	hsa_circ_0005174
novel_circ_004655	6	0	-17.9425507	0.03127344	novel
novel_circ_004675	22	11	-1.140759701	0.035230821	hsa_circ_0085465
novel_circ_004703	6	0	-17.9425507	0.03127344	novel
novel_circ_004705	0	8	18.2168285	0.007823441	novel
novel_circ_004719	4	18	2.0291653	0.007226519	hsa_circ_0002520
novel_circ_004727	3	16	2.274277798	0.007563544	hsa_circ_000883
novel_circ_004741	6	0	-17.9425507	0.03127344	hsa_circ_0007762
novel_circ_004766	0	11	18.67626012	0.00097925	novel
novel_circ_004773	6	0	-17.9425507	0.03127344	hsa_circ_0003232
novel_circ_004787	9	0	-18.5275132	0.003913285	novel
novel_circ_004790	0	7	18.02418342	0.01564141	hsa_circ_0005793
novel_circ_004816	0	7	18.02418342	0.01564141	hsa_circ_0008573
novel_circ_004892	9	0	-18.5275132	0.003913285	hsa_circ_0004805
novel_circ_004905	26	59	1.04144363	0.00224225	hsa_circ_002020
novel_circ_004937	6	0	-17.9425507	0.03127344	hsa_circ_0008794
novel_circ_004938	11	1	-3.60019132	0.006362167	hsa_circ_0072979
novel_circ_004944	0	6	17.801791	0.03127344	novel
novel_circ_004947	20	7	-1.655332874	0.012601225	hsa_circ_001472
novel_circ_004972	0	7	18.02418342	0.01564141	novel
novel_circ_005015	4	15	1.766130894	0.030957355	hsa_circ_001872
novel_circ_005032	6	0	-17.9425507	0.03127344	hsa_circ_0006521
novel_circ_005077	16	6	-1.555797201	0.034791993	hsa_circ_000586
novel_circ_005102	0	6	17.801791	0.03127344	novel
novel_circ_005151	0	6	17.801791	0.03127344	novel
novel_circ_005155	9	25	1.333171487	0.024425681	hsa_circ_0004243
novel_circ_005170	7	0	-18.16494312	0.01564141	novel
novel_circ_005176	6	0	-17.9425507	0.03127344	novel
novel_circ_005203	33	17	-1.097690979	0.015473282	hsa_circ_0003270
novel_circ_005225	0	7	18.02418342	0.01564141	hsa_circ_0047927
novel_circ_005228	0	7	18.02418342	0.01564141	hsa_circ_0046092
novel_circ_005251	0	7	18.02418342	0.01564141	hsa_circ_0034693

novel_circ_005282	1	8	2.859240299	0.039104693	hsa_circ_002038
novel_circ_005284	0	8	18.2168285	0.007823441	hsa_circ_0087421
novel_circ_005329	1	13	3.559680017	0.00183717	hsa_circ_0008619
novel_circ_005371	0	7	18.02418342	0.01564141	hsa_circ_0008741
novel_circ_005372	8	0	-18.3575882	0.007823441	novel
novel_circ_005386	5	19	1.785239717	0.010665387	hsa_circ_0007813
novel_circ_005402	0	6	17.801791	0.03127344	hsa_circ_002000
novel_circ_005405	6	0	-17.9425507	0.03127344	hsa_circ_0002687
novel_circ_005436	22	6	-2.015228819	0.002332015	novel
novel_circ_005474	0	6	17.801791	0.03127344	hsa_circ_0003967
novel_circ_005475	0	6	17.801791	0.03127344	hsa_circ_0036345
novel_circ_005480	12	2	-2.725722202	0.012969461	hsa_circ_0007755
novel_circ_005495	1	14	3.666595221	0.00183717	hsa_circ_0005638
novel_circ_005505	6	0	-17.9425507	0.03127344	hsa_circ_0003545
novel_circ_005513	2	11	2.318671917	0.022502846	hsa_circ_0046263
novel_circ_005528	0	11	18.67626012	0.00097925	hsa_circ_000935
novel_circ_005530	17	39	1.057179676	0.014587267	hsa_circ_0017586
novel_circ_005531	1	11	3.318671917	0.006362167	hsa_circ_0003164
novel_circ_005558	12	0	-18.9425507	0.000489894	hsa_circ_0006141
novel_circ_005584	10	1	-3.462687796	0.011740243	hsa_circ_0008678
novel_circ_005597	1	9	3.0291653	0.021515144	hsa_circ_0008826
novel_circ_005619	0	6	17.801791	0.03127344	hsa_circ_0005918
novel_circ_005620	6	0	-17.9425507	0.03127344	novel
novel_circ_005643	0	10	18.53875659	0.001957522	hsa_circ_0017731
novel_circ_005650	6	0	-17.9425507	0.03127344	hsa_circ_0088427
novel_circ_005663	2	11	2.318671917	0.022502846	hsa_circ_0004182
novel_circ_005707	9	1	-3.310684703	0.021515144	hsa_circ_0012553
novel_circ_005753	3	15	2.181168394	0.012762169	novel
novel_circ_005776	15	3	-2.462687796	0.004442811	novel
novel_circ_005806	15	5	-1.725722202	0.026681361	novel
novel_circ_005823	6	0	-17.9425507	0.03127344	hsa_circ_0003277
novel_circ_005852	6	0	-17.9425507	0.03127344	novel
novel_circ_005869	7	0	-18.16494312	0.01564141	hsa_circ_0003911
novel_circ_005874	18	7	-1.503329781	0.029061761	hsa_circ_0007773
novel_circ_005891	6	0	-17.9425507	0.03127344	hsa_circ_001940
novel_circ_005909	8	1	-3.140759701	0.039104693	hsa_circ_0001997
novel_circ_005915	6	0	-17.9425507	0.03127344	hsa_circ_0005330
novel_circ_005918	9	1	-3.310684703	0.021515144	hsa_circ_0008796
novel_circ_005919	0	10	18.53875659	0.001957522	hsa_circ_0058174
novel_circ_005922	9	0	-18.5275132	0.003913285	novel

novel_circ_005923	8	0	-18.3575882	0.007823441	hsa_circ_0003051
novel_circ_005930	1	8	2.859240299	0.039104693	hsa_circ_0007487
novel_circ_005959	0	7	18.02418342	0.01564141	novel
novel_circ_005961	6	22	1.733709417	0.005957079	hsa_circ_0040188
novel_circ_005976	0	6	17.801791	0.03127344	novel

Table S2. Sequences of oligonucleotides used in this study.

siRNA duplex	sense（5'-3'）	antisense（5'-3'）
circCIMT-1	CGCGGGACUAGGGAGAAUATT	UAUUCUCCCUAGUCCCGCGTT
circCIMT-2	GGGACUAGGGAGAAUAAUCTT	GAUUAUUCUCCCUAGUCCCTT

siRNA duplex	sense（5'-3'）	antisense（5'-3'）
APEX1-1	GCCCACUCAAAGUUUCUUATT	UAAGAAACUUUGAGUGGGCTT
APEX1-2	GCUGGUAACAGCAUAUGUATT	UACAUAUGCUGUUACCAGCTT
APEX1-3	GCAGUGAUCACUGUCCUAUTT	AUAGGACAGUGAUCACUGCTT

Table S3. Primers used for quantitative qRT-PCR (F: forward; R: reverse)

Gene Name	Species	Sequence (5'-3')
novel_circ_004453	Human	F:TGTCCAATTGTCGGGTTCAGG
	Human	R:TGAGGAGCTGGTCATTTTGG
novel_circ_004401(circCIMT)	Human	F:CTCATTGCGCGGGACTAGG
	Human	R:CTTGAGCTGTCTCAGTCGCA
novel_circ_003641	Human	F:TTTGAACCCAGTAAGGGAGCA
	Human	R:ACAAGTGGGACTTTGGTGGC
novel_circ_004938	Human	F:TCCCTTCCCCCTGCAATATG

	Human	R:AAGAAGCAGCTGGATTTTGCTC
novel_circ_005584	Human	F:GCTGATGAGTCTCAAGCCGT
	Human	R:AACAGCGCATTGCCTTTCAC
novel_circ_002788	Human	F:CTGGTGGAAGATCCATGAGGC
	Human	R:TGCTGTACCACGGGAAAGAT
novel_circ_005918	Human	F:GGCAGGAATTTGGGAATGAGG
	Human	R:ACAGTGATGCTTGAACCCTCT
novel_circ_005495	Human	F:CACCCTTGTTGCACACTCCA
	Human	R:TTATGGGACAAGTTCAGGCCC
novel_circ_005329	Human	F:CGATAGTTACAGCCCTGGTGG
	Human	R:GGGTCCTTGAGTCAGTCCTCT
novel_circ_002435	Human	F:AAGAGCCAGGATGTTGCCG
	Human	R:ATTATGGAAAGGCCGGGTGAG
novel_circ_002790	Human	F:TGGTGAGGATTCAGTTGGAGTG
	Human	R:TTCCCGGTAAGCACTCTGTT
novel_circ_002126	Human	F:GGGACTCATGTGGCATTTCTC
	Human	R:CAAGCTTGTGCTGTTCGCT
novel_circ_004327	Human	F:TTTTGGTCTCTGGAGTGCCTG
	Human	R:GCAGCAGTACAGCCATTTGTT
novel_circ_001944	Human	F:AGGAAGGTTGCTTAGCCAGAC
	Human	R:CTGCCTTTTCCAAAGCGTGT
novel_circ_001973	Human	F:GCAGACGGTGATGCTTTCAG
	Human	R:AGCATTGAGGCCAGTCTTGA

linear 004401	Human	F:TGCTGTAGGGGAGACCAAGA
	Human	R:ATTTGGGTAGGCAGCATCTCT
Gsk3b	Human	F:GAGGTGCAAAACGGAGCAAC
	Human	R:CAATGCCAGATCCCCAGGAA
Notch2	Human	F:TATTAGTGGGGGCCTCTGGG
	Human	R:TCCTCTTCTGCCTGCCTTTG
Pik3cb	Human	F:TCTGACTCCCAGCCTTTTCAC
	Human	R:TACACCCCTGATTCACACCC
Myc	Human	F:ATGAGGGGCTGTGTTTAGAGG
	Human	R:CCCAAAGCAAAGCACATTCCC
Cdkn1a	Human	F:ACTCTCAGGGTCGAAAACGG
	Human	R:AAGGGCCTGGCATAATGAACA
Smad3	Human	F:CTTCCCATCCCGACACACTT
	Human	R:GTGAGTCACTGCGTTTCTGC
circCIMT	Mouse	F:GTGGAAGCAGCGGAGGATAC
	Mouse	R:TTTTACACGCTTCCGCCAAC
Gsk3b	Mouse	F:CCCTTAGCAGATGGCCTGTT
	Mouse	R:CAAGTACACCACTCACCGCA
Notch	Mouse	F:CACAGCAGTGTCTCTCTCACTT
	Mouse	R:ACAAACTACCGCACAGAGAGAA
Pik3cb	Mouse	F:GTCTGAAACCGGAGAGCCC
	Mouse	R:CGAAGGCAGCAAGAGACTGG
Myc	Mouse	F:TGGCTTATCTTTCAGCTCCATCC

	Mouse	R:CTGGTGTTGGGGGAGTGTTG
Cdkn1a	Mouse	F:GTTGGGGGATCTACCCTCTC
	Mouse	R:CTAAGGCCGAAGATGGGGAA
Smad3	Mouse	F:TCACTGAATGTGCCTAGAGCC
	Mouse	R:AGGCGACTAGTTGGCTATGTG

Table S4. Probes of circCIMT

Primer	Sequence (5' to 3')
circCIMT-5'biotin	TAACGCGCCCTGATCCCTCTTATTAGTACCCGGTC
